# Accessible and reproducible mass spectrometry imaging data analysis in Galaxy

**DOI:** 10.1093/gigascience/giz143

**Published:** 2019-12-09

**Authors:** Melanie Christine Föll, Lennart Moritz, Thomas Wollmann, Maren Nicole Stillger, Niklas Vockert, Martin Werner, Peter Bronsert, Karl Rohr, Björn Andreas Grüning, Oliver Schilling

**Affiliations:** 1 Institute of Surgical Pathology, Medical Center – University of Freiburg, Breisacher Straße 115a, 79106 Freiburg, Germany; 2 Faculty of Biology, University of Freiburg, Schänzlestraße 1, 79104 Freiburg, Germany; 3 Biomedical Computer Vision Group, BioQuant, IPMB, Heidelberg University, Im Neuenheimer Feld 267, 69120 Heidelberg, Germany; 4 Institute of Molecular Medicine and Cell Research, Faculty of Medicine, University of Freiburg, Stefan-Meier-Straße 17, 79104 Freiburg, Germany; 5 Faculty of Medicine - University of Freiburg, Breisacher Straße 153, 79110 Freiburg, Germany; 6 Tumorbank Comprehensive Cancer Center Freiburg, Medical Center – University of Freiburg, Breisacher Straße 115a, 79106 Freiburg, Germany; 7 German Cancer Consortium (DKTK) and Cancer Research Center (DKFZ), Hugstetter Straße 55, 79106 Freiburg, Germany; 8 Department of Computer Science, University of Freiburg, Georges-Köhler-Allee 106, 79110 Freiburg, Germany

**Keywords:** mass spectrometry imaging, MALDI imaging, spatially resolved mass spectrometry, proteomics, metabolomics, Galaxy, computational workflows, reproducibility

## Abstract

**Background:**

Mass spectrometry imaging is increasingly used in biological and translational research because it has the ability to determine the spatial distribution of hundreds of analytes in a sample. Being at the interface of proteomics/metabolomics and imaging, the acquired datasets are large and complex and often analyzed with proprietary software or in-house scripts, which hinders reproducibility. Open source software solutions that enable reproducible data analysis often require programming skills and are therefore not accessible to many mass spectrometry imaging (MSI) researchers.

**Findings:**

We have integrated 18 dedicated mass spectrometry imaging tools into the Galaxy framework to allow accessible, reproducible, and transparent data analysis. Our tools are based on Cardinal, MALDIquant, and scikit-image and enable all major MSI analysis steps such as quality control, visualization, preprocessing, statistical analysis, and image co-registration. Furthermore, we created hands-on training material for use cases in proteomics and metabolomics. To demonstrate the utility of our tools, we re-analyzed a publicly available N-linked glycan imaging dataset. By providing the entire analysis history online, we highlight how the Galaxy framework fosters transparent and reproducible research.

**Conclusion:**

The Galaxy framework has emerged as a powerful analysis platform for the analysis of MSI data with ease of use and access, together with high levels of reproducibility and transparency.

## Background

Mass spectrometry imaging (MSI) is increasingly used for a broad range of biological and clinical applications because it allows the simultaneous measurement of hundreds of analytes and their spatial distribution. The versatility of MSI is based on its ability to measure many different kinds of molecules such as peptides, metabolites, or chemical compounds in a large variety of samples such as cells, tissues, fingerprints, or human-made materials [1–5]. Depending on the sample, the analyte of interest, and the application, different mass spectrometers are used [6]. The most common ionization sources are MALDI (matrix-assisted laser desorption/ionization), desorption electrospray ionization, and secondary ion mass spectrometry. Typical mass analyzers are time-of-flight (TOF) devices and ion traps.

Owing to the variety of samples, analytes, and mass spectrometers, MSI is suitable for highly diverse use cases ranging from plant research to (pre-)clinical, pharmacologic studies, and forensic investigations [2, 7–9]. On the other hand, the variety of research fields hinders harmonization and standardization of MSI protocols. Recently efforts were started to develop optimized sample preparation protocols and show their reproducibility in multicenter studies [10–13]. In contrast, efforts to make data analysis standardized and reproducible are in their infancy.

Reproducibility of MSI data analyses is hindered by the common use of software with restricted access such as proprietary software, license-requiring software, or unpublished in-house scripts [14]. Open source software has the potential to advance accessibility and reproducibility issues in data analysis but requires complete reporting of software versions and parameters, which is not yet routine in MSI [15–17].

At the same time, the introduction of the open standard file format imzML has opened new avenues to the community and an increasing number of open source software tools are emerging [18]. Yet, many of these tools necessitate steep learning curves, in some cases even requiring programming knowledge to make use of their full range of functions [19–23].

To overcome problems with accessibility of software and computing resources, standardization, and reproducibility, we developed MSI data analysis tools for the Galaxy framework that are based on the open source software suites Cardinal [21], MALDIquant [20], and scikit-image [24]. Galaxy is an open source computational platform for biomedical research that was developed to support researchers without programming skills with the analysis of large datasets, e.g., in the field of next-generation sequencing. Galaxy is used by hundred thousands of researchers and provides thousands of different tools for many different scientific fields [25].

### Aims

With the present publication, we aim to raise awareness within the MSI community of the advantages being offered by the Galaxy framework with regard to standardized and reproducible data analysis pipelines. Second, we present newly developed Galaxy tools and offer them to the MSI community through the graphical front-end and “drag-and-drop” workflows of the Galaxy framework. Third, we apply the MSI Galaxy tools to a publicly available dataset to study N-glycan identity and distribution in murine kidney specimens to demonstrate use of a Galaxy-based MSI analysis pipeline that facilitates standardization and reproducibility and is compatible with the principles of FAIR (findable, accessible, interoperable, and re-usable) data and MIAPE (minimum information about a proteomics experiment) [26, 27].

### The Galaxy framework for flexible and reproducible data analysis

In essence, the Galaxy framework is characterized by 4 hallmarks: (i) use of a graphical front end that is web browser based, hence alleviating the need for advanced information technology skills or the requirement to locally install and maintain software tools; (ii) access to large-scale computational resources for academic users; (iii) provenance tracking and full version control, including the ability to switch between software and tool version and to publish complete analysis, thus enabling full reproducibility; (iv) access to a vast array of open source tools with the ability to seamlessly pass data from one tool to another, thus generating added value by interoperability.

Multiple Galaxy servers on essentially every continent provide access to large computing resources, data storage capabilities, and hundreds of pre-installed tools for a broad range of data analysis applications through a web browser–based graphical user interface [28–30]. Additionally, there are >100 public Galaxy servers available that offer more specific tools for niche application areas. For local use, Galaxy can be installed on any computer ranging from private laptops to high-performance computing clusters. So-called “containers” exist, which facilitate a fully functional 1-click installation independent of the operating system. Hence, local Galaxy servers are easily deployed even in “private” network situations in which these servers remain invisible and inaccessible to outside users. This ability empowers Galaxy for the analysis of sensitive and protected data, e.g., in a clinical setting.

In the Galaxy framework, data analysis information is stored alongside the results of each analysis step to ensure reproducibility and traceability of results. The information includes tool names, versions, and all other parameters that are necessary to capture the provenience of an experiment [31].

We propose that MSI research can greatly benefit from the possibility to privately or publicly share data analysis histories, workflows, and visualizations with collaboration partners or the entire scientific community, e.g., as online supplementary data for peer-reviewed publications. The latter step easily fulfills the criteria of the suggested MSI minimum reporting guidelines [6, 16].

The Galaxy framework is predestined for the analysis of multi-omics studies because it facilitates the integration of software of different origin into 1 analysis [32, 33]. The possibility of seamlessly linking tools of different origins has outstanding potential for MSI studies, which often rely on different software platforms to analyze MSI data, additional MS/MS data (from liquid chromatography coupled tandem mass spectrometry [LC-MS/MS]), and (multimodal) imaging data. As a result of community-driven efforts, >100 tools for proteomic and metabolomics data analysis are readily available in Galaxy [34–38]. Increasing integration of MSI with other omics approaches such as genomics and transcriptomics is anticipated, and the Galaxy framework offers a powerful and future-proof platform to tackle complex, interconnected data-driven experiments.

## Findings

### The newly available MSI toolset in the Galaxy framework

**Figure 1:**
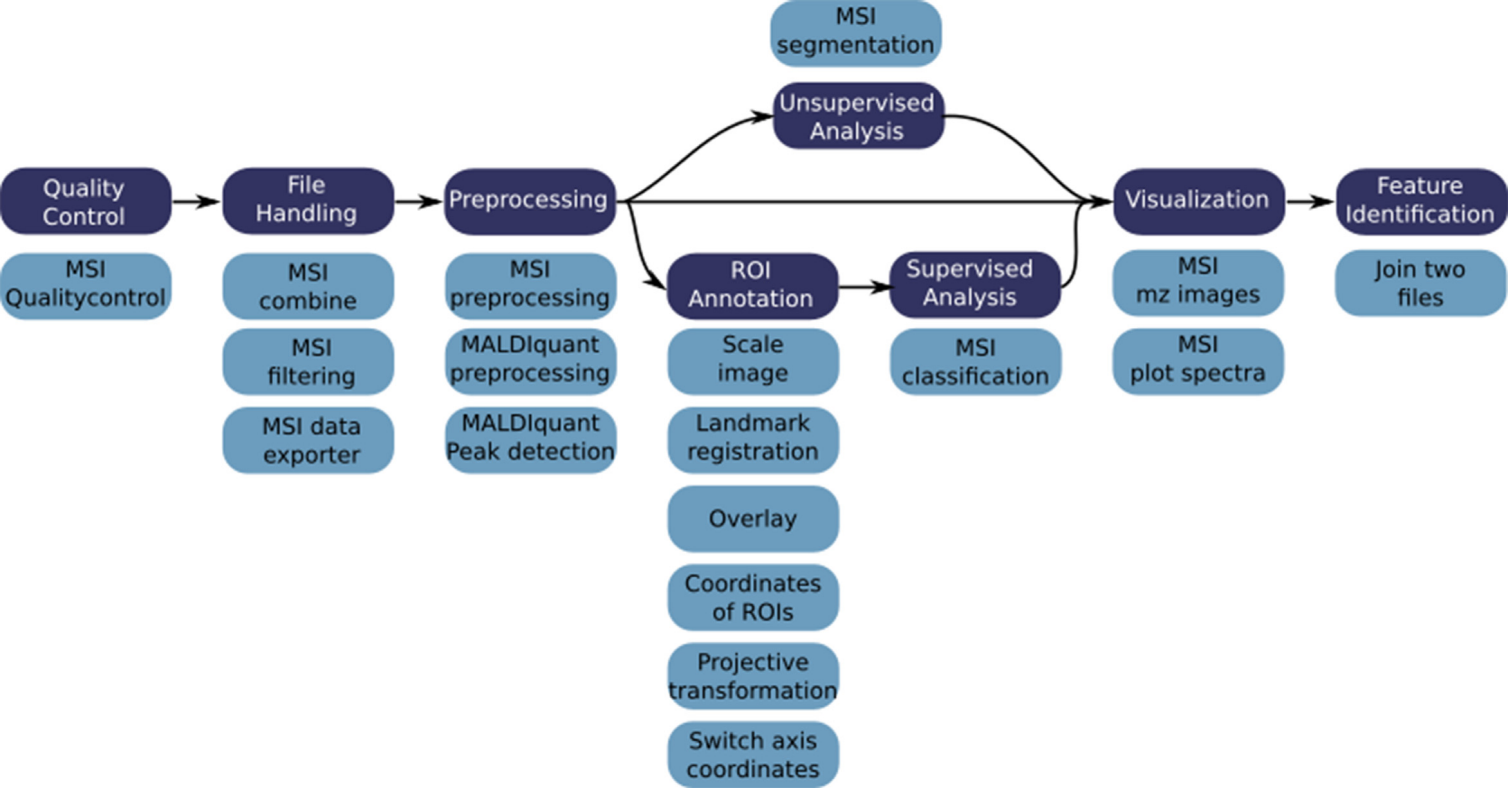
Typical MSI data analysis steps and newly developed Galaxy tools. Typical MSI data analysis steps include quality control, file handling, preprocessing, ROI annotation, supervised and unsupervised statistical analysis, visualizations, and identification of features. Owing to the variety of MSI applications, tools of all or only a few of these categories are used and the order of use is highly flexible. To serve a broad range of data analysis tasks, we provide 18 tools that cover all common data analysis procedures and can be arbitrarily connected to allow customized analysis. Dark blue: MSI data analysis steps; pale blue: newly developed Galaxy tools.

We have developed 18 Galaxy tools that are based on the commonly used open source software packages Cardinal, MALDIquant, and scikit-image and enable all steps that commonly occur in MSI data analysis (Fig. 1) [20, 21, 24]. In order to deeply integrate those tools into the Galaxy framework, we developed bioconda packages and biocontainers, as well as a so-called wrapper for each tool [31, 39]. The MSI tools consist of R scripts that were developed on the basis of Cardinal and MALDIquant functionalities, extended for more analysis options and a consistent framework for input and output of metadata (Additional File 1). Cardinal and MALDIquant are well-established R packages and are commonly used open source software for the analysis of MSI data [40–45]. Cardinal is under active development and provides a multitude of processing and analysis options for MSI data [46]. MALDIquant was originally developed for the analysis of classical MALDI-TOF data but offers powerful preprocessing options that are applicable for the analysis of MSI data [44, 45]. The image-processing tools that are part of the region of interest (ROI) annotation (co-registration) workflow are built from scratch using functionality from the scikit-image library. Scikit-image is an open source image-processing library for Python. All tools are deliberately built in a modular way to enable highly flexible analysis and to allow a multitude of additional functionalities by combining the MSI-specific tools with already available Galaxy tools.

#### Data formats and data handling

We extended the Galaxy framework to support open and standardized MSI data files such as imzML, which is the default input format for the Galaxy MSI tools. Today, the major mass spectrometer vendors directly support the imzML standard and several tools exist to convert different file formats to imzML [47]. Data can be easily uploaded to Galaxy via a web browser or via a built-in FTP functionality. Intermediate result files can be further processed in the interactive environment that supports R Studio and Jupyter or downloaded for additional analysis outside of Galaxy [48].

To facilitate the parallel analysis of multiple files, the Galaxy framework offers so-called dataset collections. Numerous files can be represented in a dataset collection, allowing simultaneous analysis of all files while the effort for the user is similar as for single files. MSI metadata such as spectra annotations, *m/z*lists, and statistical results are stored as tab-separated values (TSV) files, thus enabling processing by a plethora of tools both inside and outside the Galaxy framework. All graphical results of the MSI tools are stored as concise vector graphic PDF reports with publication-quality images.

#### Quality control and visualization tools

##### MSI Quality control

**Figure 2:**
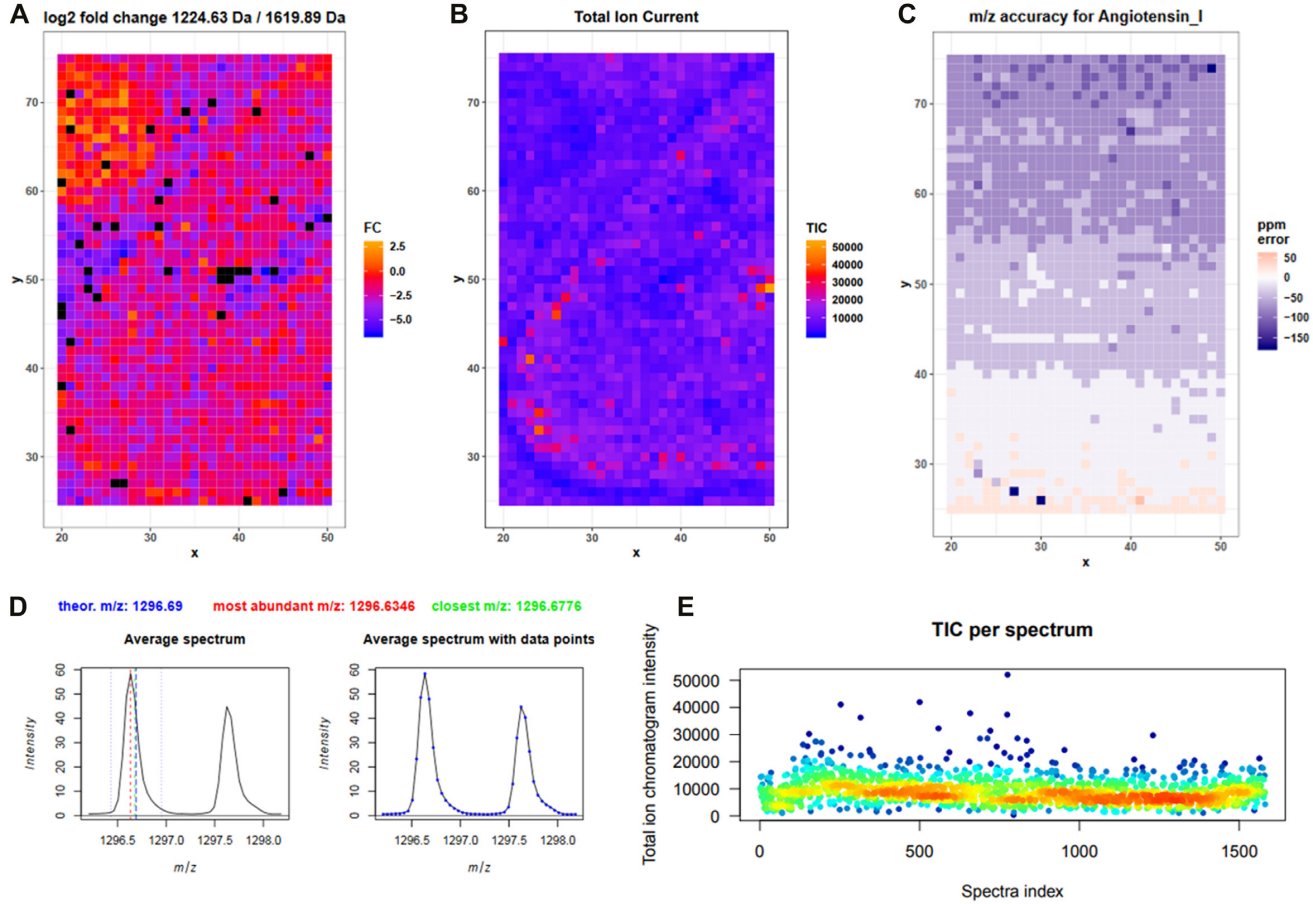
Exemplary quality control plots. The quality control report contains >30 different plots, 5 of which are shown here. They derive from the peptide imaging in mouse kidney training that we provide in the Galaxy training network [58]. **A**, Control of tryptic digestion with a bombesin spot next to the tissue. The log_2_ fold change of the cleaved vs uncleaved bombesin peptide shows the digestion efficiency in the spot. **B**, Total ion current (TIC) in each pixel. **C**, Accuracy of *m/z* for the internal calibrant angiotensin I in each pixel. **D**, Average mass spectrum showing the angiotensin I peak and its first isotope together with vertical lines indicating the theoretical *m/z* of angiotensin (blue), the most abundant *m/z* in the chosen window (red), and the closest *m/z* value in the file (green). **E**, TIC for each spectrum.

Quality control is an essential step in data analysis and should be used not only to judge the quality of the raw data but also to control processing steps such as smoothing, peak picking, and intensity normalization. Therefore, we have developed the “MSI Qualitycontrol” tool, which automatically generates a comprehensive PDF report with >30 different plots that enable a global view of all aspects of the MSI data including intensity distribution, *m/z* accuracy, and segmentation maps (Fig. 2). For example, poor-quality spectra, such as those with low total ion current or low number of peaks, can be directly spotted in the quality report and subsequently be removed by applying the “MSI data exporter” and “MSI filtering” tools.

##### MSI mz image

**Figure 3:**
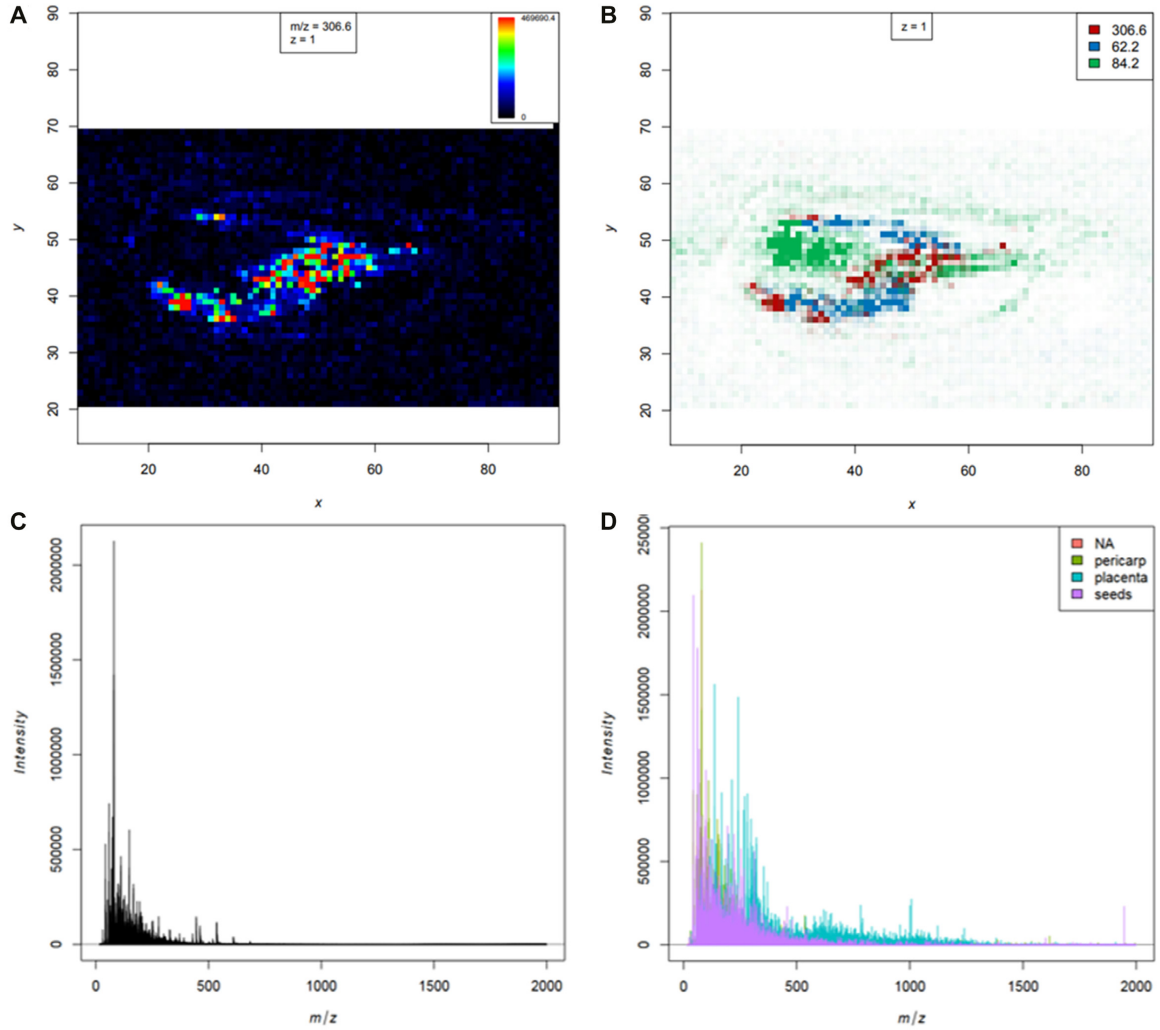
Visualization of heat maps and mass spectra. Visualizations of heat maps, overlay image, and mass spectra plots of a chilli section that is part of the chilli training that we provide in the Galaxy training network [19, 58]. **A**, Heat map of the *m/z* feature 306.6 that corresponds to capsaicin. **B**, Overlay of the *m/z* features 306.6, 62.2, and 84.2 that show different distribution in the chilli section. **C**, Average mass spectrum for the complete chilli dataset. **D**, Overlay of single mass spectra that belong to different chilli compartments: green: pericarp; light blue: placenta; purple: seeds; red: average mass spectra of all other chilli spectra.

The “MSI mz image” tool allows the automatic generation of a publication-quality PDF file with distribution heat maps for all *m/z*features provided in a TSV file. Contrast enhancement and smoothing options are available, as well as the possibility of overlaying several *m/z* features in 1 image (Fig. 3A and B).

##### MSI plot spectra

The “MSI plot spectra” tool displays multiple single or average mass spectra in a PDF file. Overlay of multiple single or averaged mass spectra with different colors in 1 plot is also possible (Fig. 3C and D).

The Galaxy framework already offers various visualization options for TSV files, including heat maps, barplots, scatterplots, and histograms. This enables a quick visualization of the properties of TSV files obtained during MSI analysis.

#### MSI file-handling tools

A large variety of tools that allow for filtering, sorting, and manipulating of TSV files is already available in Galaxy and can be integrated into the MSI data analysis. Some dedicated tools for imzML file handling were newly integrated into the Galaxy framework.

##### MSI combine

The “MSI combine” tool allows several imzML files to be combined into a merged dataset. This is especially important to enable direct visual but also statistical comparison of MSI data that derived from multiple files. With the “MSI combine tool,” individual MSI datasets either are placed next to each other in a coordinate system or can be shifted in the *x* or *y* direction in a user-defined way. The output of the tool contains a single file with the combined MSI data and an additional TSV file with spectra annotations; i.e., each spectrum is annotated with its original file name (before combination) and, if applicable, with previously defined annotations such as diagnosis, disease type, and other clinical parameters.

##### MSI filtering

The “MSI filtering” tool provides options to filter *m/z* features and pixel (spectra) of interest, either by applying manual ranges (minimum and maximum *m/z*, spatial area as defined by *x/y* coordinates) or by keeping only *m/z* features or coordinates of pixels that are provided in a TSV file. Unwanted *m/z* features such as predefined contaminant features can be removed within a preselected *m/z* tolerance.

##### MSI data exporter

The “MSI data exporter” can export the spectra, intensity, and *m/z* data of an imzML file together with their summarized properties into TSV files.

#### Region of interest annotation tools

**Figure 4:**
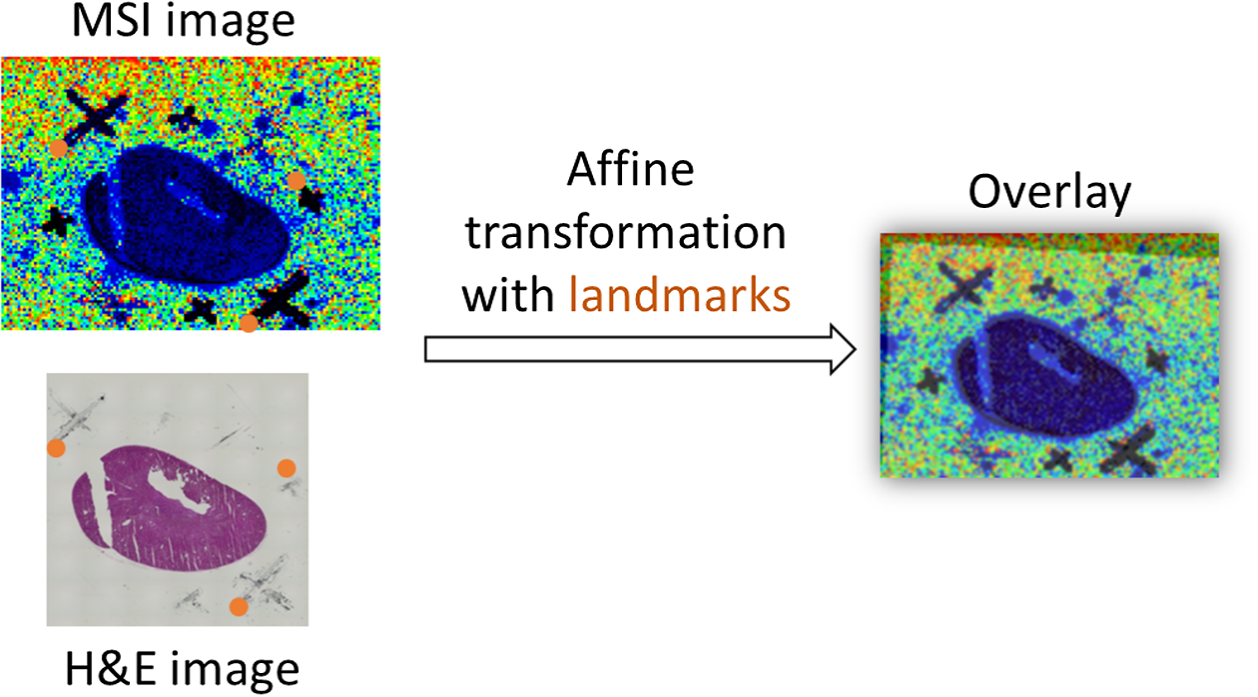
Co-registration strategy using affine transformation and landmarks. Co-registration of an MSI image with a histological hematoxylin-eosin (H&E) image from a mouse kidney via affine transformation. The affine transformation is estimated from the landmarks (red dots) that the user has to provide. Landmarks can be either characteristic marks of the sample or marks applied outside the tissue, e.g., with a xylene-resistant pen (black crosses). The transformation matrix, estimated by the affine transformation estimation tool, can be used to generate an overlay of both images (right image) for visual inspection. Moreover, the matrix can be used to transfer the annotated regions of interest in the H&E image to the MSI coordinates (not shown here).

For supervised analysis, spatial ROI can be defined. Those are commonly annotated on a photograph or histological image that shows the morphological features of the sample. We extended and developed 6 new Galaxy tools and combined them with existing tools into a workflow that enables co-registration of the real image (photograph or histological image), ROIs, and the MSI image by alignment using an affine transformation [49]. The transformation is estimated by a least-squares method using landmarks from both real and MSI image that are annotated outside Galaxy, for example, using the GNU Image Manipulation Program (GIMP) (Fig. 4) [50]. For more robust estimation of the transformation, random sample consensus is used on random subsets of landmark pairs [51].

The co-registration workflow includes 6 newly developed Galaxy tools, as follows.

##### Scale Image

The "scale image" tool can resize an image relative to the original image or using absolute dimensions with nearest neighbor, bilinear, or bicubic interpolation.

##### Landmark registration

The "landmark registration" tool estimates the affine transformation between 2 sets of points using the random sample consensus [51].

##### Overlay

The "overlay" tool overlays 2 images, transforming 1 using a transformation matrix. The tool can be used to visually asses the performance of the registration.

##### Coordinates of ROI

The "coordinates of ROI" tool extracts the indices of all pixels of an ROI from a binary image.

##### Projective transformation points

The "projective transformation points" tool applies a transformation matrix to a set of points.

##### Switch axis coordinates

The "switch axis coordinates" tool can be used to change the origin of a set of points in a coordinate system.

In the supporting information, we also provide automated workflows to convert annotation files from proprietary Bruker software (spotlist.txt and regions.xml) into annotation files that are compatible with the Galaxy MSI tools.

#### Preprocessing tools

Preprocessing of raw MSI spectra is performed to reduce data size and to remove noise, inaccuracies, and biases to improve downstream analysis. Crucial steps are peak picking to reduce file size and remove noise features, intensity normalization to make spectra within and between different samples comparable, as well as *m/z* recalibration to improve comparability and identification of analytes. We have developed 3 dedicated MSI preprocessing tools that are based on a variety of preprocessing algorithms from both the Cardinal and MALDIquant packages. An overview of all available preprocessing options is available in Additional File 2.

##### MSI preprocessing

The “MSI preprocessing” tool offers a multitude of algorithms that are useful to preprocess raw MSI data: intensity normalization to the total ion current (TIC), baseline removal, smoothing, peak picking, peak alignment, peak filtering, intensity transformation, binning, and resampling.

##### MALDIquant preprocessing and MALDIquant peak detection

Both MALDIquant tools offer a multitude of preprocessing algorithms that complement those of the Cardinal-based MSI preprocessing tool such as *m/z* re-calibration, peak picking on average mass spectra, and picking of mono-isotopes.

#### Statistical analysis tools

A multitude of statistical analysis options for TSV files is already available in Galaxy; the most MSI-relevant tools are from the Workflow4metabolomics project and consist of unsupervised and supervised statistical analysis tools [52]. For specific purposes of spatially resolved MSI data analysis, we have integrated Cardinal's powerful spatially aware statistical analysis options into the Galaxy framework.

##### MSI segmentation

The “MSI segmentation” tool enables spatially aware unsupervised statistical analysis with principal component analysis, spatially aware *k*-means clustering, and spatial shrunken centroids [53, 54].

##### MSI classification

The “MSI classification” tool offers 3 options for spatially aware supervised statistical analysis: partial least squares (discriminant analysis), orthogonal partial least squares (discriminant analysis), and spatial shrunken centroids [53].

#### Analyte identification tools

Determination of *m/z* on its own often remains insufficient to identify analytes. Compound fragmentation and tandem mass spectrometry are typically used for compound identification by mass spectrometry. In MSI, the required local confinement of the mass spectrometry analysis severely limits the compound amounts that are available for fragmentation. Hence, direct on-target fragmentation is rarely used in MSI. A common practice for compound identification includes a combinatorial approach in which LC-MS/MS data are used to identify the analytes while MSI analyzes their spatial distribution. This approach requires assigning putative analyte information to *m/z*values within a given accuracy range.

##### Join 2 files on a column allowing a small difference

The "Join 2 files on a column allowing a small difference" tool allows for the matching of numeric columns of 2 TSV files on the smallest distance, which can be absolute or in ppm. This tool can be used to identify the *m/z* features of a TSV file by matching them to already identified *m/z* features of another TSV file (e.g., from a database or from an analysis workflow).

Community efforts such as Galaxy-M, Galaxy-P, Phenomenal, and Workflow4Metabolomics have led to a multitude of metabolomics and proteomics analysis tools that are available in Galaxy today [34–38]. These tools enable the analysis of additional tandem mass spectrometry data that are often acquired to aid identification of MSI *m/z* features. Databases to which the results can be matched, such as UniProt and LIPID MAPS, are directly available in Galaxy [55, 56]. The highly interdisciplinary and modular data analysis options in Galaxy render it a very powerful platform for MSI data analyses that are part of a multi-omics study.

### Accessibility and training

All described tools are easily accessible and usable via the European Galaxy server [29]. Furthermore, all tools are deposited in the Galaxy Toolshed from which they can be easily installed into any other Galaxy instance (Additional File 3) [57]. We have developed bioconda packages and biocontainers that allow for version control and automated installation of all tool dependencies—those packages are also useful outside Galaxy to enhance reproducibility [31, 39]. For researchers who do not want to use publicly available Galaxy servers, we provide a prebuilt Docker image that is easy to install independent of the operating system.

For a swift introduction into the analysis of MSI data in Galaxy, we have developed training material for metabolomics and proteomic use cases and deposited it to the central repository of the Galaxy Training Network [58, 59]. The training materials consist of a comprehensive collection of small example datasets, step-by-step explanations, and workflows that enable any interested researcher to follow the training and understand it through active participation.

The first training explains data upload in Galaxy and describes the quality control of mouse kidney tissue section in which peptides were imaged with an old MALDI-TOF [60]. The dataset contains peptide calibrants that allow the control of the digestion efficiency and *m/z* accuracy. Export of MSI data into TSV files and further filtering of those files is explained as well.

The second training explains the examination of the spatial distribution of volatile organic compounds in a chilli section. The training roughly follows the corresponding publication and explains how average mass spectra are plotted and only the relevant *m/z* range is kept, as well as how to automatically generate many *m/z* distribution maps and overlay several *m/z* feature maps [19].

The third training determines and identifies N-linked glycans in mouse kidney tissue sections with MALDI-TOF and additional LC-MS/MS data analysis [61, 62]. The training covers combining datasets, preprocessing as well as unsupervised and supervised statistical analysis to find potential N-linked glycans that have different abundances in the PNGase F–treated kidney section compared to the kidney section that was treated with buffer only. The training further covers identification of the potential N-linked glycans by matching their *m/z* values to a list of N-linked glycan *m/z* that were identified by LC-MS/MS. The full dataset is used as a case study in the following section.

### Case study

**Figure 5:**
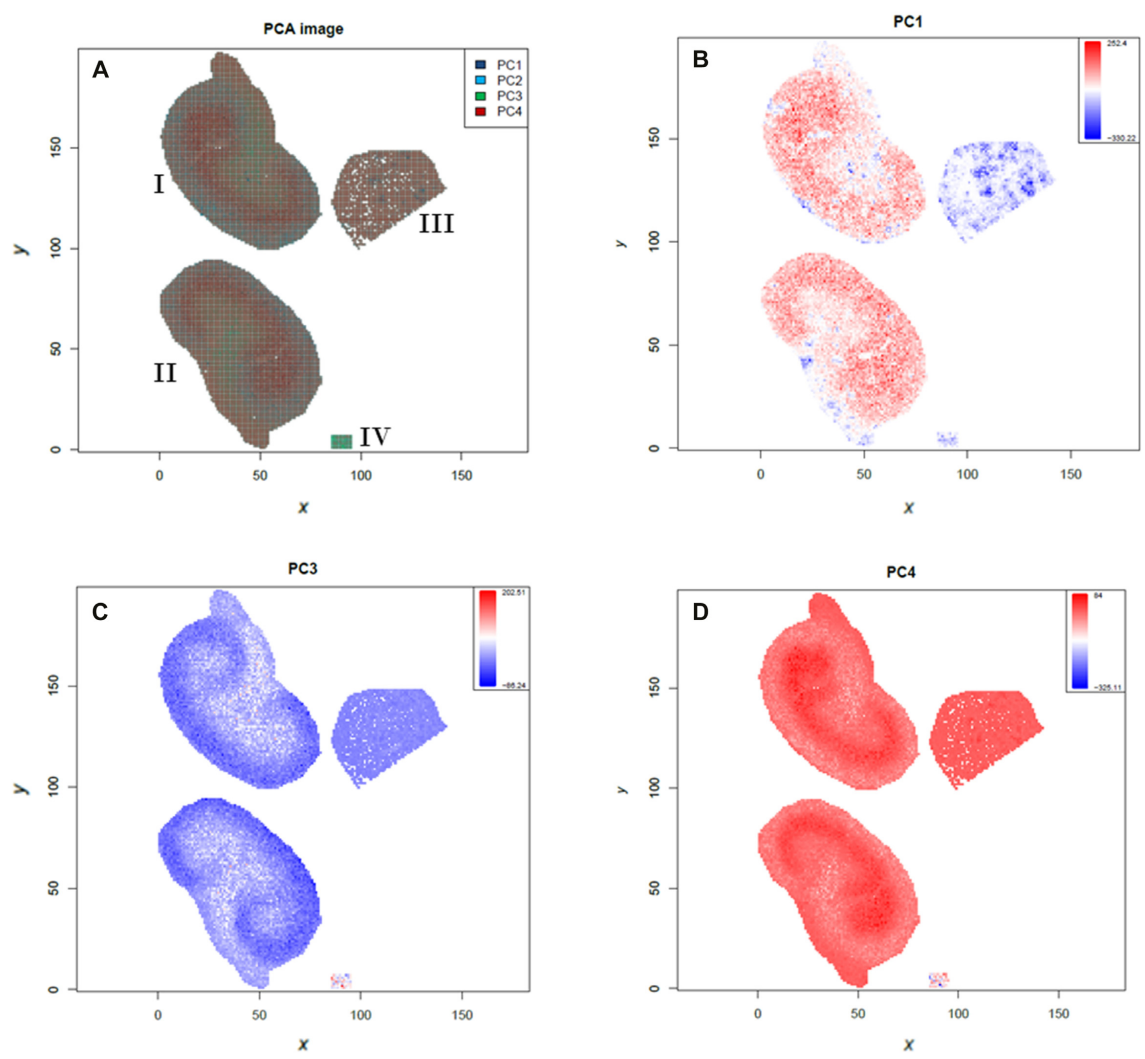
Results from the unsupervised statistical analysis of N-linked glycans in murine kidney tissues. Principal component analysis (PCA) of treated, control, and calibrant files. **A**, Overlay of all principal component (PC) scores. **B–D**, Principal components 1, 3, and 4 that discriminate treated and control tissue or different kidney compartments. I = treated kidney1, II = treated kidney2, III = control kidney, IV = calibrants.

**Figure 6:**
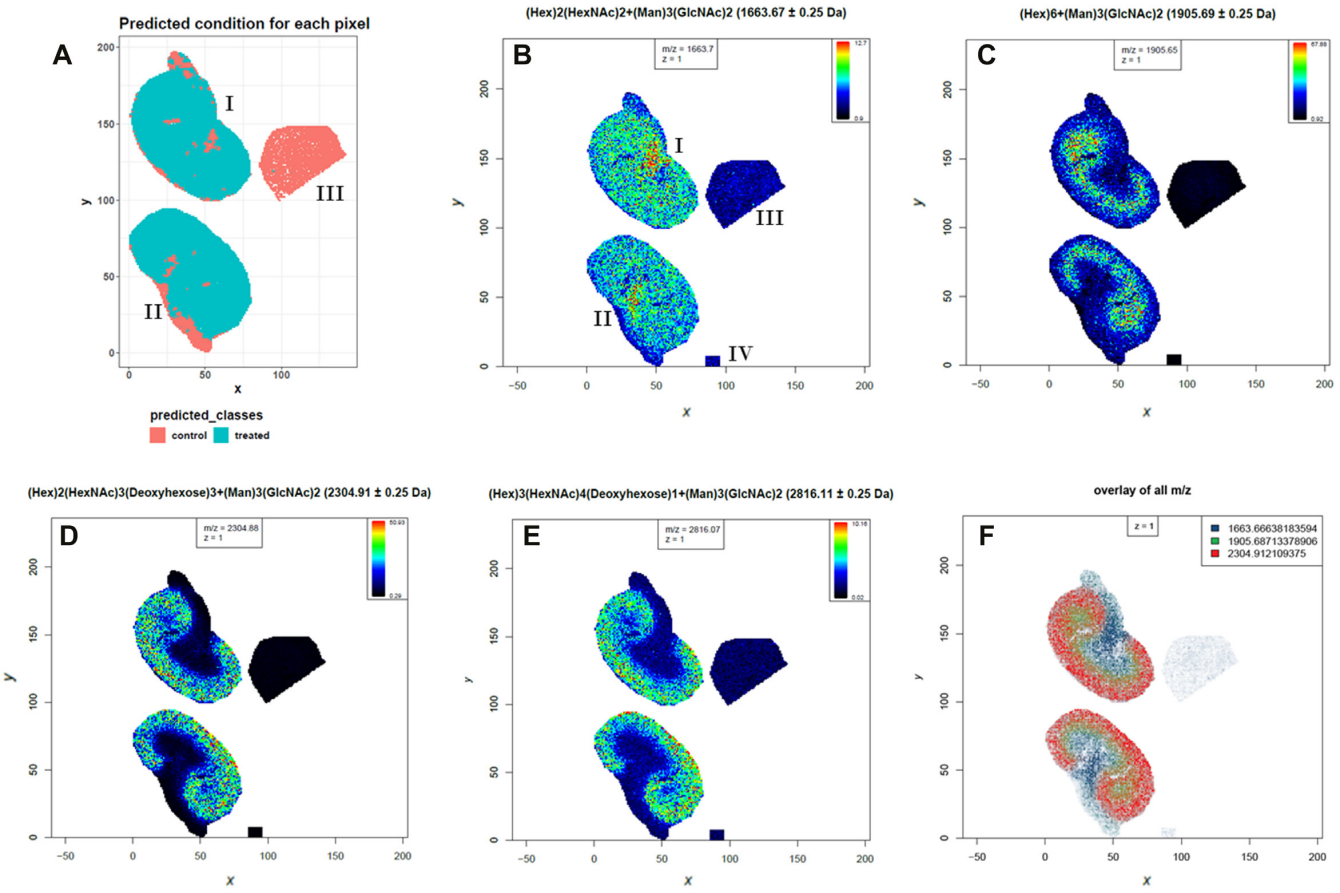
Results from the supervised statistical analysis of N-linked glycans in murine kidney tissues. The supervised spatial shrunken centroids method was used to determine *m/z* features that are more abundant in the treated than in the control tissue specimen. **A**, Spatial shrunken centroids class prediction for all spectra. **B–E**, Intensity distribution images for 4 identified N-linked glycans (Hex)_2_(HexNAc)_2_(deoxyhexose)_1_+(Man)_3_(GlcNAc)_2_ (*m/z* 1,663.6), (Hex)_6_+(Man)_3_(GlcNAc)_2_ (*m/z* 1,905.7), (Hex)_2_(HexNAc)_3_(deoxyhexose)_3_+(Man)_3_(GlcNAc)_2_ (*m/z* 2,304.9), and (Hex)_3_(HexNAc)_4_(deoxyhexose)_1_+(Man)_3_(GlcNAc)_2_ (*m/z* 2,816.0). **F**, Overlay of 3 N-linked glycans with different distribution in the kidney. The ion distribution images and the overlay image were generated with contrast enhancement by suppression on TIC-normalized data. I = treated kidney1, II = treated kidney2, III = control kidney, IV = calibrants.

To exemplify the utility of our MSI tools we re-analyzed the N-glycan dataset that was recently made available by Gustafsson et al. via the PRIDE repository with accession PXD009808 [62, 63]. The aim of the study was to demonstrate that their automated sample preparation method for MALDI imaging of N-linked glycans successfully works on formalin-fixed paraffin-embedded (FFPE) murine kidney tissue [61]. PNGase F was printed on 2 FFPE murine kidney sections to release N-linked glycans from proteins while in a third section 1 part of the kidney was covered with N-glycan calibrants and another part with buffer to serve as a control. The tissues were measured with a MALDI-TOF/TOF mass spectrometer and a spatial resolution of 100 µm that leads to oversampling of the 250-µm PNGase F array [61]. We downloaded all 4 imzML files (2 treated kidneys, control and calibrants) from PRIDE and uploaded them with the composite upload function into Galaxy. To obtain an overview of the files we used the “MSI Qualitycontrol” tool. We resampled the *m/z* axis, combined all files, and reran the “MSI Qualitycontrol” tool to directly compare the 4 subfiles (Additional File 4). Next, we performed TIC normalization, smoothing, and baseline removal by applying Cardinal algorithms [21]. Spectra were aligned to the stable peaks that are present in ≥80% of all spectra [64]. Spectra in which <2 stable peaks could be aligned were removed. This affected mainly spectra from the control file. Peak picking, detection of mono-isotopic peaks, and binning were performed on the average spectra of each subfile [64]. The obtained *m/z* features were extracted with Cardinal's “peaks” algorithm from the normalized, smoothed, baseline-removed and aligned file. Next, principal component analysis with 4 components was performed (Fig. 5) [21]. To find potential N-linked glycans, the 2 treated tissues were compared to the control tissue with the supervised spatial shrunken centroids algorithm [53]. Spatial shrunken centroids is a multivariate classification method that was specifically developed to account for the spatial structure of the data (Fig. 6A) [53]. The supervised analysis provided us with 28 *m/z* features that discriminated between the 2 PNGase F–treated kidneys and the control kidney with a spatial shrunken centroids *P*-value < 0.05 and higher abundance in the treated kidneys. Mapping those features to N-glycans reported in the original publication ([61], Supplementary Table S2) revealed the identity of 16 N-glycans with an average *m/z* error of 49 ppm (Table 1). Fifteen of those N-glycans match to the findings of the original publication. Whilst our workflow did not identify the reported N-glycan at 1,647.635 *m/z*, an additional N-glycan at 1,542.62 *m/z* was found. The intensity distribution for 4 N-glycans on the TIC-normalized dataset is depicted in Fig. 6B–E, and 3 of them are overlaid in Fig. 6F.

**Table 1:** N-linked glycans identified in the re-analysis

*m/z*	Centers	*t-*Statistics	Adjusted *P*-values	M+Na+	Composition	ppm
1,257.47424	38.24	51.97	0	1,257.41	(Hex)_2_+(Man)_3_(GlcNAc)_2_	51
1,743.68713	32.11	48.56	0	1,743.57	(Hex)_5_+(Man)_3_(GlcNAc)_2_	67
1,419.55334	40.68	48.2	0	1,419.47	(Hex)_3_+(Man)_3_(GlcNAc)_2_	59
1,905.68713	48.61	44.78	0	1,905.63	(Hex)_6_+(Man)_3_(GlcNAc)_2_	30
2,304.91211	43.53	42.36	0	2,304.83	(Hex)_2_(HexNAc)_3_(deoxyhexose)_3_+(Man)_3_(GlcNAc)_2_	36
1,850.71216	25.3	42.01	0	1,850.65	(Hex)_1_(HexNAc)_3_(deoxyhexose)_1_+(Man)_3_(GlcNAc)_2_	34
1,581.62573	18.07	40.64	0	1,581.53	(Hex)_4_+(Man)_3_(GlcNAc)_2_	61
1,809.72461	10.81	38.15	0	1,809.63	(Hex)_2_(HexNAc)_2_(deoxyhexose)_1_+(Man)_3_(GlcNAc)_2_	52
2,158.88721	14.77	38.03	0	2,158.77	(Hex)_2_(HexNAc)_3_(deoxyhexose)_2_+(Man)_3_(GlcNAc)_2_	54
1,663.66638	10.27	32.26	0	1,663.57	(Hex)_2_(HexNAc)_2_+(Man)_3_(GlcNAc)_2_	58
1,688.71509	8.68	28.29	0	1,688.61	(HexNAc)_3_(deoxyhexose)_1_+(Man)_3_(GlcNAc)_2_	62
1,485.62378	8.67	26.89	0	1,485.53	(HexNAc)_2_(deoxyhexose)_1_+(Man)_3_(GlcNAc)_2_	63
2,012.78394	7.3	26.72	0	2,012.71	(Hex)_2_(HexNAc)_3_(deoxyhexose)_1_+(Man)_3_(GlcNAc)_2_	37
2,816.11206	6.92	26.35	0	2,816.01	(Hex)_3_(HexNAc)_4_(deoxyhexose)_1_+(Man)_3_(GlcNAc)_2_	36
2,067.75903	5.69	14.52	0	2,067.67	(Hex)_7_+(Man)_3_(GlcNAc)_2_	43
1,542.61902	5.59	8.08	0	1,542.55	(HexNAc)_3_+(Man)_3_(GlcNAc)_2_	45

We could identify 16 N-linked glycans by matching the *m/z* features of the MSI data (col. 1) to the identified *m/z* features of the LC-MS/MS experiment (col. 5). We allowed a maximum tolerance of 300 ppm and multiple matches. Only single matches occurred with an average *m/z* error of 46 ppm (col. 6). Centers, t-statistics and adjusted p-values obtained by the spatial shrunken centroid algorithm are reported in column 2-4. Glycan composition in column 6: Hex: Hexose, Man: Mannose, GlcNAc: N-Acetyl-D-glucosamine, HexNAc: N-Acetyl-D-hexosamine

The complete analysis was performed in the European Galaxy instance with MSI tools based on Cardinal version 1.12.1 and MALDIquant 1.18 [21, 29]. Despite having used different algorithms for preprocessing and statistical analysis, we reached similar findings as compared to [61]. The reproducibility of the results shows the capacity of our pipeline. To enable full “methods reproducibility” we provide the analysis history and workflow in this publication as supporting information. Those can be easily published on the Galaxy platform and provide more information than requested by the minimum reporting guidelines MSI MIAPE and MIAMSIE (minimum information about a mass spectrometry imaging experiment) [6, 16]. The Galaxy software itself but also the shared histories and workflows fulfill the FAIR principles [27].

## Conclusions

With the integration of the MSI data analysis toolset, we have incorporated an accessible and reproducible data analysis platform for MSI data in the Galaxy framework. Our MSI tools complement the multitude of already available Galaxy tools for proteomics and metabolomics that are maintained by Galaxy-M, Galaxy-P, Phenomal, and Workflow4Metabolomics [34–38]. We are in close contact with those communities and would like to encourage developers of the MSI community to join forces and make their tools available in the Galaxy framework. We currently focused on reproducible and accessible data analysis, but we are planning to integrate interactive visualizations, more support for very large files, and more tools for specific use cases into the Galaxy framework. Last, we would like to invite the MSI community to use the advantages of the Galaxy framework to advance MSI data analysis.

## Availability of Supporting Source Code and Requirements

Project name: Mass spectrometry imaging workbench in Galaxy

RRID number:SCR_017410 (https://scicrunch.org/resolver/RRID:SCR_017410)

Project homepage: https://github.com/galaxyproteomics/tools-galaxyp and https://github.com/BMCV/galaxy-image-analysis

Galaxy Toolshed: https://toolshed.g2.bx.psu.edu/

Operating system(s): Unix (platform independent with Docker)

Training repository: https://galaxyproject.github.io/training-material/ “mass spectrometry imaging” tutorials can be found in the sections “metabolomics” and “proteomics.”

Docker image: https://github.com/foellmelanie/docker-galaxy-msi

License: MIT

## Availability of Supporting Data and Materials

Galaxy workflow to convert Bruker ROI.xml files: https://usegalaxy.eu/u/melanie-foell/w/msi-workflow-bruker-xml-conversion-to-tabular-file

Galaxy workflow to convert Bruker spotlists: https://usegalaxy.eu/u/melanie-foell/w/bruker-spotlist-conversion-to-tabular-file

Galaxy workflow co-registration: https://usegalaxy.eu/u/melanie-foell/w/co-registration-of-msi-image-and-real-image-with-landmarks

Galaxy workflow N-linked glycans re-analysis: https://usegalaxy.eu/u/melanie-foell/w/msi-workflow-complete-n-glycan-analysis

Galaxy history N-linked glycans re-analysis: https://usegalaxy.eu:/u/melanie-foell/h/re-analysis-of-pride-dataset-pxd009808—maldi-imaging-of-n-linked-glycans-in-murine-kidney-specimens

Archival copies of the code and workflows are available from the *GigaScience* GigaDB repository [65].

## Additional Files

**Additional File 1:** Overview of R-functions in the MSI tools. For each Galaxy MSI tool the R-functions that do not belong to the basic R-package are listed.

**Additional File 2:** Overview of available preprocessing options

**Additional File 3:** Collection of direct links to the toolshed location for each tool

**Additional File 4:** Exemplary quality control plots for the combined N-glycan imaging file

giz143_GIGA-D-19-00158_Original_Submission

giz143_GIGA-D-19-00158_Revision_1

giz143_Response_to_Reviewer_Comments_Original_Submission

giz143_Reviewer_1_Report_Original_Submission Chris Armit -- 5/27/2019 Reviewed

giz143_Reviewer_1_Report_Revision_1 Chris Armit -- 9/23/2019 Reviewed

giz143_Reviewer_2_Report_Original_Submission Ralph Weber -- 6/4/2019 Reviewed

giz143_Reviewer_3_Report_Original_Submission Jane Armstrong -- 6/13/2019 Reviewed

giz143_Supplemental_Additional_Files

## Abbreviations

FAIR: findable, accessible, interoperable, and re-usable; FFPE: formalin-fixed paraffin-embedded; FTP: file transfer protocol; H&E: hematoxylin-eosin; LC-MS/MS: liquid chromatography tandem mass spectrometry; MALDI: matrix-assisted laser desorption/ionization; MIAMSIE: minimum information about a mass spectrometry imaging experiment; MIAPE: minimum information about a proteomics experiment; MSI: mass spectrometry imaging; PRIDE: proteomics identifications; ROI: region of interest; TIC: total ion current; TOF: time of flight; TSV: tab-separated values.

## Competing Interests

The authors declare that they have no competing interests.

## Funding

O.S. acknowledges support by the German Research Council (DFG, GR 1748/6-1, SCHI 871/8-1, SCHI 871/9-1, SCHI 871/11-1, SCHI 871/12-1, INST 39/900-1, and SFB850-Project Z1 (INST 39/766-3), RO-5694/1-1), the German-Israel Foundation (Grant No. I-1444-201.2/2017), and the European Research Council (780730, ProteaseNter, ERC-2017-PoC). B.A.G. is supported by the German Federal Ministry of Education and Research (031L0101C de.NBI-epi) and the European Open Science Cloud (EOSC-Life) (Grant No. 824087). K.R. acknowledges support of the Federal Ministry of Education and Research (de.NBI, CancerTelSys) and the German Research Foundation (SFB 1129, RTG 1653). The article processing charge was funded by the German Research Foundation (DFG) and the University of Freiburg in the funding programme Open Access Publishing.

## Authors’ Contributions

M.C.F. developed the MSI Galaxy tool wrappers, the training material, and the case study. L.M. acquired data for the training material, tested MSI tools and training material, and provided useful feedback. T.W. developed the Galaxy tools and workflow for co-registration and contributed to build the Galaxy tool wrappers. M.N.S. tested MSI tools, co-registration tools, and training material and provided useful feedback. N.V. built the Galaxy tool wrappers for the co-registration tools and tested them. M.W., P.B., K.R., B.A.G., and O.S. contributed to the conceptualization, methodology, and funding acquisition. B.A.G. integrated the MSI file formats into Galaxy, contributed to build the training material and tool wrappers, and integrated all tool wrappers into Galaxy. O.S. and M.C.F. wrote the manuscript. All authors critically read and approved the manuscript's contents.
