# Supplementary material for: Accessible and reproducible mass spectrometry imaging data analysis in Galaxy
Source: Gigascience. 2019 Dec 9;8(12):giz143. doi: 10.1093/gigascience/giz143 (PMC6901077; doi:10.1093/gigascience/giz143)
Supplement: giz143_GIGA-D-19-00158_Revision_1 [file giz143_giga-d-19-00158_revision_1.pdf]

# GigaScience


# Accessible and reproducible mass spectrometry imaging data analysis in Galaxy

--Manuscript Draft--

| | |
|---|---|
| **Manuscript Number:** | GIGA-D-19-00158R1 |
| **Full Title:** | Accessible and reproducible mass spectrometry imaging data analysis in Galaxy |
| **Article Type:** | Technical Note |
| **Funding Information:** | Deutsche Forschungsgemeinschaft (GR 1748/6-1, SCHI 871/8-1, SCHI 871/9-1, SCHI 871/11-1, SCHI 871/12-1, INST 39/900-1, and SFB850-Project Z1 (INST 39/766-3), RO-5694/1-1) — Dr Oliver Schilling<br>German-Israeli Foundation for Scientific Research and Development (I-1444-201.2/2017) — Dr Oliver Schilling<br>European Research Council (780730, ProteaseNter, ERC-2017-PoC) — Dr Oliver Schilling<br>Bundesministerium für Bildung und Forschung (031L0101C de.NBI-epi)) — Dr Bjoern Gruening<br>European Open Science Cloud (824087) — Dr Bjoern Gruening<br>Bundesministerium für Bildung und Forschung (de.NBI, CancerTelSys) — Prof Karl Rohr<br>Deutsche Forschungsgemeinschaft (SFB 1129, RTG 1653) — Prof Karl Rohr |
| **Abstract:** | Background: Mass spectrometry imaging is increasingly used in biological and translational research as it has the ability to determine the spatial distribution of hundreds of analytes in a sample. Being at the interface of proteomics/metabolomics and imaging, the acquired data sets are large and complex and often analyzed with proprietary software or in-house scripts, which hinder reproducibility. Open source software solutions that enable reproducible data analysis often require programming skills and are therefore not accessible to many MSI researchers. Findings: We have integrated 18 dedicated mass spectrometry imaging tools into the Galaxy framework to allow accessible, reproducible, and transparent data analysis. Our tools are based on Cardinal, MALDIquant, and scikit-image and enable all major MSI analysis steps such as quality control, visualization, preprocessing, statistical analysis, and image co-registration. Further, we created hands-on training material for use cases in proteomics and metabolomics. To demonstrate the utility of our tools, we re-analyzed a publicly available N-linked glycan imaging dataset. By providing the entire analysis history online, we highlight how the Galaxy framework fosters transparent and reproducible research. Conclusion: The Galaxy framework has emerged as a powerful analysis platform for the analysis of MSI data with ease of use and access together with high levels of reproducibility and transparency. |
| **Corresponding Author:** | Melanie Christine Föll<br><br>GERMANY |
| **Corresponding Author Secondary Information:** | |
| **Corresponding Author's Institution:** | |
| **Corresponding Author's Secondary Institution:** | |
| **First Author:** | Melanie Christine Föll |
| **First Author Secondary Information:** | |
| **Order of Authors:** | Melanie Christine Föll |
| | |


*Powered by Editorial Manager® and ProduXion Manager® from Aries Systems Corporation*

| | |
|---|---|
| | Lennart Moritz |
| | Thomas Wollmann |
| | Maren Stillger |
| | Niklas Vockert |
| | Martin Werner |
| | Peter Bronsert |
| | Karl Rohr |
| | Bjoern Gruening |
| | Oliver Schilling |
| **Order of Authors Secondary Information:** | |
| **Response to Reviewers:** | We sincerely thank the reviewers for their thoughtful comments and inspections of our manuscript. We have improved the manuscript by carefully addressing the raised concerns and provide a point-by-point response below. In the manuscript, we have highlighted paragraphs with major changes in blue to allow an easier tracking of the changes.<br><br>Reviewer #1 comment 1:<br>This Technical Note describes software tools for mass spectrometry image (MSI) analysis and integration of these tools into Galaxy. This enables reproducible MSI analysis to be accomplished in the context of a graphical user interface. The manuscript is well written and the authors should be commended for ensuring that the software tools are all open source.<br>In Figure 1 the authors highlight all 18 open source software tools in the MSI analysis toolkit. However, in the manuscript detailed descriptions are only provided for 12 of the software tools. I would like to see descriptions for the following 6 software tools: 1) Scale image; 2) Landmark registration; 3) Overlay; 4) Coordinates of ROIs; 5) Projective transformation; 6) Switch axis coordinates. This would be of great benefit to the end user who may wish to reuse these software tools. It would be additionally helpful if these 6 tools were listed in the supplementary file (supp-1.xlsx), which currently only lists 12 Galaxy tools.<br>>> Authors:<br>We thank the reviewer for pointing out this inconsistency, which has now been corrected by adding descriptions of the imaging tools in the main text and also added their specifications in supplementary file 1.<br><br>Reviewer #1 comment 2:<br>In the section entitled "Accessibility & training" the authors state that, "all tools are deposited in the Galaxy Toolshed from where they can be easily installed into any other Galaxy instance". Could the authors please specify where in the Galaxy Toolshed I would find all 18 software tools? The link that the authors provide (https://toolshed.g2.bx.psu.edu/) does not bring me to a directory that lists all 18 tools. Furthermore, I cannot see any mass spectrometry tools listed on this page. A direct link to the 18 software tools would be far more helpful and would encourage reuse of the toolkit.<br>>> Authors:<br>We agree with the reviewer that direct toolshed links for each tool are more helpful. Therefore we added a supplementary file with the direct links for all 18 tools.<br>Reviewer #1 comment 3:<br>As proof-of-principle, the authors used a publicly available N-linked glycan imaging dataset as a test dataset. The authors acknowledged that annotation was "infeasible on the MSI images" and they describe a method whereby the researcher annotates an adjacent histological section and then co-registers the two images using an affine transform. I was intrigued by this approach but I was unable to find the resolution of the MS images in the manuscript. Furthermore, the authors did not provide any detail of pixel size, or even scale bars, for the murine kidney test data set that is shown in Figures 2 and 3. However, I did find these details in the original publications that describe these data where it is stated that MSI profiling utilised a "centre to centre spacing of 250µm". (Gustafsson et al., 2018, Data Brief. 21: 185-188; Gustafsson et |

al., 2015, Anal Bioanal Chem. 407(8): 2127-2139). Should we infer from this that the pixel dimensions are approximately 250µm x 250µm? I would like some clarity from the authors on the resolution of the MS images.
>> Authors:

We sincerely apologize for not being clear in the spatial resolution of the N-glycan dataset. We have extended the text to address the issue. The PNGase F was spotted with 250µm centre to centre spacing but the data was acquired by oversampling with a spatial resolution of 100µm leading to a pixel size of 100µm (Reference Gustafsson et al. 2015, page 2130).

Reviewer #1 comment 4:
Whereas I can see great value in the software tools, I would question the utility of this low-resolution MSI dataset. A pixel resolution of 250µm x 250µm is fairly coarse. Consequently, it would be impossible to determine from these data whether the MS profiles reflect N-linked glycan distribution in key microanatomical compartments of the kidney such as the proximal tubule, juxtaglomerular apparatus, or podocyte. I was wondering whether the authors have used their software tools on cellular-resolution MSI data? From a biological perspective, this would be far more interesting. In this respect, the NIH/NIDDK-funded GUDMAP Consortium have delivered a whole series of publications that detail gene expression in the kidney at the microanatomical level (Brunskill et al., 2008, Dev Cell. 15(5):781-91; Lindström et al., 2018, J Am Soc Nephrol. 29(3):785-805; Adam et al., 2017, Development. 144(19):3625-3632). I was wondering whether the authors would consider using their toolkit to analyse more biologically relevant, single-cell resolution MSI datasets? This would be of greater interest to the research community and would be an improved means of highlighting the reuse potential of this toolkit.
>> Authors:
We fully understand the concerns of the reviewer but have to apologize that commercial mass spectrometers and available protocols do not yet allow for single cell analysis. The reviewer refers to gene expression dataset with single-cell resolution. However, such datasets do not exists in the field of MSI; a limitation that is due to technical shortcomings of the method. Technological progress may remove this limitation in the future, but as of today, most MSI experiments are performed with a spatial resolution between 30 and 250 µm. The actual resolution is dependent on the purpose of the experiment and limited by the technical capabilities of the mass spectrometer and the limited amount of analytes that can be extracted from a single cell. While the technical improvements aim towards enabling cellular resolution, data analysis steps are mainly working on single pixel (mass spectra) and therefore only affected by the increasing file size as more pixels will be measured to cover the same sample area. Therefore, the limitation for single cell analysis lies not in the analysis tools but in the method itself. To show the usefulness of our tools, we also re-analyze volatile metabolites in a chilli section and the peptide distribution in a mouse kidney; both are part of the accompanying training material. Hence, we respectfully decline further case study analysis.

Reviewer #2 comment 1:
The authors have integrated three different mass spectrometry imaging (MSI) software packages (Cardinal, MALDIquant, and scikit-image) into the Galaxy framework. The Galaxy tools and workflows presented cover a wide range of common MSI data processing and analysis steps, and allow Proteomics and Metabolomics researchers to build useful and flexible workflows. It is evident that a substantial amount of work has gone not just into wrapping the different MSI packages into the Galaxy Framework, but also in developing and writing the online training material and use cases. The manuscript is well written and is relevant to the scope of the journal. The manuscript could benefit from revision as indicated in the detailed comments listed below.
Page 4 - "Galaxy is used by hundred thousands of researchers and provides thousands of different tools for many different scientific fields." - Include more realistic and accurate statistics on users and tools. The reference cited [25, Afgan et al.] does provide some specific numbers / statistics on tools and users.

Powered by Editorial Manager® and ProduXion Manager® from Aries Systems Corporation

| | |
|---|---|
| | >> Authors:<br><br>We did not provide specific numbers for two reasons: 1) The specific numbers do not reflect the number of researchers using Galaxy, as they only refer to the main Galaxy server, neglecting the european, australian and the many smaller servers. 2) The specific numbers were collected about one year ago and are already outdated and will be even more outdated when the manuscript will be read in the future.<br>Reviewer #2 comment 2:<br>Page 6 - Change "All tools are deliberately build" to "all tools are deliberately built"<br>>> Authors:<br>Done as suggested.<br><br>Reviewer #2 comment 3:<br>Page 6 – Change "cleverly combining" to "combining"<br>>> Authors:<br>Done as suggested.<br><br>Reviewer #2 comment 4:<br>Page 6 - Please, add a short paragraph to section "The newly available MSI toolset in the Galaxy Framework" that described the status of the different packages (i.e. Cardinal, MALDIquant, etc) - for example, are they all commonly used tools? Well established? Cutting edge?<br>>> Authors:<br>Done as suggested.<br><br>Reviewer #2 comment 5:<br>Page 7 - "Nowadays" too informal and not specific - past n years? Add a reference to back up statement.<br>>> Authors:<br><br>We changed the sentence accordingly and reference 43 lists current vendor imzML compatible software and imzML converting software.<br><br>Reviewer #2 comment 6:<br>Page 7 - "file collections" - Should this be "dataset collections" (i.e. "official" galaxy terminology)? If so, change throughout the manuscript.<br>>> Authors:<br>Done as suggested.<br><br>Reviewer #2 comment 7:<br>Page 7 - "Numerous files can be represented in a file collection allowing simultaneous analysis of all files while the effort for the user is the same as for a single file". This is somewhat misleading. Whilst dataset collections can be very useful in Galaxy and in some cases allow parallel processing to be performed, in an optimised or user friendly manner, they often require an additional step to prepare or merge the data.<br>>> Authors:<br><br>That's true, our sentence was misleading. We changed it into "while the effort for the user is similar as for single files".<br>Reviewer #2 comment 8:<br>Page 7 - Add more information on imzML format for the reader<br>>> Authors:<br><br>As the imzML format is the community standard, we thought this information is not useful in the manuscript. The file information that is relevant for successfully handling this file type are described in more detail in the training material.<br><br>Reviewer #2 comment 9:<br>Page 8: Change "Spectra with bad quality" to "poor quality spectra"<br>>> Authors:<br>Done as suggested.<br><br>Reviewer #2 comment 10:<br>Page 10 - "However, annotation of these ROIs is infeasible on the MSI images" explain |

Powered by Editorial Manager® and ProduXion Manager® from Aries Systems Corporation

why
>> Authors:
Done as suggested.

Reviewer #2 comment 11:
Page 10 - Add a reference for "Random sample consensus (RANSAC)"
>> Authors:
Done as suggested.

Reviewer #2 comment 12:
Page 11 - "Join two files on a column allowing a small difference:" - Is this the real name of the tools. Convoluted name for a tool.
>> Authors:

Yes, it's the full name, we thought that it's better to be descriptive than short for our users.

Reviewer #2 comment 13:
Page 14 - Change "Matching those features that potentially are N-glycans to the m/z list of the original publication" to "mapping features to N-glycans reported in the original publication"
>> Authors:
Done as suggested.

Reviewer #2 comment 14:
Page 14 - Change "While we missed the 1647.635 m/z N-glycan, we found another N-glycan with 1542.62 m/z." to "whilst our workflow did not identify the reported N-glycan at 1647.635 m/z, an additional N-glycan at 1542.62 m/z was found."
>> Authors:
Done as suggested.

Reviewer #2 comment 15:
Page 16 - "In Gustafsson's own terms from a recent publication, our results show that their results are reproducible, because we, as another group, have followed as closely as possible their data analysis procedure and arrived at similar results [16].The reproducibility of the results shows the capacity of our pipeline. To enable what Gustafsson has described as "methods reproducibility" we provide the complete analysis history and the corresponding workflow. With this in hand, any other researcher can use the same tools and parameters in Galaxy to obtain the same result as we did." Overly wordy, therefore rephrase.
>> Authors:
We agree that this paragraph was confusing, therefore we rewrote it to make it easier to read and understand.

Reviewer #2 comment 16:
Page 17 - "Publishing histories and workflows from Galaxy requires only a few clicks" - too informal. Rephrase, in particular the words "a few clicks".
>> Authors:
We changed the sentence accordingly.

Reviewer #2 comment 17:
Preprocessing (Page 10-14)
Provide a short description for TIC normalisation, baseline removal and smoothing, etc, including details for the different parameters used (Supplementary information),? I.e. which algorithms and parameters for peak picking, detection of monoisotopic peaks and binning were applied? And why?
>> Authors:

The N-glycan dataset that was used to demonstrate a use-case for the workbench is also part of the Galaxy training network (https://training.galaxyproject.org/training-material/topics/metabolomics/tutorials/msi-finding-nglycans/tutorial.html).
In this tutorial we describe in detail the different analysis steps and the choice of

algorithms and parameters. The overall goal of the case study was to reproduce the original study, therefore we have chosen algorithms and parameters that most closely resembled the data analysis from the original study. A link to the training material repository is included in the manuscript, which we think is more sustainable, as we plan to extend and update the training material in the future.
For users that are interested in more details of the case study, we have published a real-world Galaxy history which provides all details about used algorithms, parameters, tool version and references.
The authors would like to stress the fact, that we think that sharing a history is more reproducible and transparent than trying to describe parameters in text.

Reviewer #2 comment 18:
Add a table that summarises the different options for each pre-processing step. What normalization and peak picking options are available? I.e. "multitude of algorithms". Provide a more detailed summary. This could be a further advantage over proprietary software having more flexibility on pre-processing steps.
>> Authors:

We added this information as additional file.

Reviewer #2 comment 19:
Is median fold change used (or available as an option) as described in the following paper? Abbassi-Ghadi, Nima, Emrys A Jones, Kirill A Veselkov, Juzheng Huang, Sacheen Kumar, Nicole Strittmatter, Ottmar Golf, et al. 2015. "Repeatability and Reproducibility of Desorption Electrospray Ionization-Mass Spectrometry (DESI-MS) for the Imaging Analysis of Human Cancer Tissue: A Gateway for Clinical Applications." Analytical Methods 7 (1): 71-80. https://doi.org/10.1039/C4AY01770F.
>> Authors:

Unfortunately, no. Not yet. For now we have focused on the implementation of Cardinal and MALDIquant functionalities that contain the most common normalization methods TIC and median. We plan to extend and update our tools if requested by users and are happy to add additional normalization methods. We encourage everyone to use the normal Galaxy communication channels like gitter and help.galaxyproject.org to get in touch with us and provide feedback.

Reviewer #2 comment 20:
Include references for the methods/approached that have been applied. For example - A detailed description for the underlying principles applied as part of MALDIquant pre-processing and MALFIquant peak detection are missing. Add the relevant references and details (add to Supplementary Information if needed). Same applies to other methods / approaches.
>> Authors:
Thanks for this recommendation. We added further references to the manuscript whenever possible. Unfortunately most of the Cardinal functions are specific methods for the Cardinal package and no further descriptions does exist except for the code in the github repository which is referenced already in our manuscript.

Reviewer #2 comment 21:
Add figures to the main text or Supplementary Information for the following:
Page 8 - Figures in the data visualization section would make for a more intuitive read - For instance - include figures for a number of the "30 different plots"
>> Authors:
Done as suggested.

Reviewer #2 comment 22:
Page 8 - Include figures for both the MSI image and MSI plot spectra (with overlay)
>> Authors:
Done as suggested.

Reviewer #2 comment 23:
Page 9 - ROI annotation tool, particularly use of "affine transformation", could be aided by a figure

Powered by Editorial Manager® and ProduXion Manager® from Aries Systems Corporation

| | |
|---|---|
| | >> Authors:<br>Done as suggested.<br><br>Reviewer #2 comment 24:<br>Page 14 - Outputs / Results "Quality Control" tool<br>>> Authors:<br>Done as suggested.<br><br>Reviewer #3 comment 1:<br>This is an interesting paper that provides new tools for the Galaxy platform and that will be of use to biomedical researchers generating and analysing MSI data. The authors demonstrate the value of the toolset that they have developed by reanalysing a published dataset and they provide open access to source code and additional training documentation. Developing open source tools is commendable and I therefore support publication.<br>Minor Revisions:<br>Section describing the new MSI toolset: Making more of a distinction in the text between the newly developed tools and existing Galaxy tools would be helpful to the reader.<br>>> Authors:<br><br>We agree with the reviewer and have made it more clear in the manuscript to distinguish between new tools and already existing tools. Moreover, the newly developed tools are depicted in Figure 1.<br>Reviewer #3 comment 2:<br>I also suggest adding 'tool(s)' to each subheading within this section i.e. 'Quality control and visualization tools:'<br>>> Authors:<br>Done as suggested.<br><br>Reviewer #3 comment 3:<br>Figure 1: The figure title should make it clear that these are the newly developed Galaxy tools: the use of 'associated' is ambiguous.<br>>> Authors:<br>Done as suggested.<br><br>Reviewer #3 comment 4:<br>The figure legend could be improved by clarifying that the MSI data analysis steps are in dark blue and the new tools are shown in pale blue.<br>>> Authors:<br>Done as suggested.<br><br>Reviewer #3 comment 5:<br>Sectioning describing Accessibility and training: I found navigation to the training sets difficult. The link to the Galaxy Training Network/repository is to the home page and the reader then has to click through several pages to find each of the 3 examples. The names are not obvious from the manuscript and I had to click through a number of other examples to find them. The training material is excellent but just difficult to find.<br>>> Authors:<br>We added the information next to the link to the training repository: mass spectrometry imaging tutorials can be found in the metabolomics and proteomics categories of the training material.<br><br>Reviewer #3 comment 6:<br>Availability of supporting data: I did not seem to have the necessary permission to access the datasets provided in the last link.<br>>> Authors:<br>We are sorry for the inconvenience. Galaxy values data privacy a lot and we made a mistake in granting everyone permissions. We updated the dataset permissions to make sure that the link works now.<br><br>Reviewer #3 comment 7:<br>Add FAIR to the list of abbreviations. |

Powered by Editorial Manager® and ProduXion Manager® from Aries Systems Corporation

| | |
|---|---|
| | >> Authors:<br>Done as suggested. |
| **Additional Information:** | |
| **Question** | **Response** |
| Are you submitting this manuscript to a special series or article collection? | No |
| **Experimental design and statistics**<br><br>Full details of the experimental design and statistical methods used should be given in the Methods section, as detailed in our Minimum Standards Reporting Checklist. Information essential to interpreting the data presented should be made available in the figure legends.<br><br>Have you included all the information requested in your manuscript? | Yes |
| **Resources**<br><br>A description of all resources used, including antibodies, cell lines, animals and software tools, with enough information to allow them to be uniquely identified, should be included in the Methods section. Authors are strongly encouraged to cite Research Resource Identifiers (RRIDs) for antibodies, model organisms and tools, where possible.<br><br>Have you included the information requested as detailed in our Minimum Standards Reporting Checklist? | Yes |
| **Availability of data and materials**<br><br>All datasets and code on which the conclusions of the paper rely must be either included in your submission or deposited in publicly available repositories (where available and ethically appropriate), referencing such data using a unique identifier in the references and in the “Availability of Data and Materials” | Yes |

| | |
|---|---|
| section of your manuscript.<br><br>Have you have met the above requirement as detailed in our Minimum Standards Reporting Checklist? | |

Powered by Editorial Manager® and ProduXion Manager® from Aries Systems Corporation

Manuscript

Click here to view linked References

Click here to access/download;Manuscript;MSI_Galaxy_revised.docx

# Title page

**Accessible and reproducible mass spectrometry imaging data analysis in Galaxy**

**Authors:**

Melanie Christine Föll [1,2], Lennart Moritz [1], Thomas Wollmann [3], Maren Nicole Stillger [1,2,4], Niklas Vockert [3], Martin Werner [1,5,6,7], Peter Bronsert [1,5,6,7], Karl Rohr [3], Björn Andreas Grüning [8], Oliver Schilling [1,5,7,9]

1. Institute of Surgical Pathology, Medical Center – University of Freiburg, Breisacher Straße 115a 79106 Freiburg, Germany
2. Faculty of Biology, University of Freiburg, Schänzlestraße 1 79104 Freiburg, Germany
3. Biomedical Computer Vision Group, BioQuant, IPMB, Heidelberg University, Im Neuenheimer Feld 267 69120 Heidelberg, Germany
4. Institute of Molecular Medicine and Cell Research, Faculty of Medicine, University of Freiburg, Stefan-Meier-Straße 17 79104 Freiburg, Germany
5. Faculty of Medicine - University of Freiburg, Germany
6. Tumorbank Comprehensive Cancer Center Freiburg, Medical Center – University of Freiburg, Germany
7. German Cancer Consortium (DKTK) and Cancer Research Center (DKFZ), Heidelberg, Germany
8. Department of Computer Science, University of Freiburg, Georges-Köhler-Allee 106 79110 Freiburg, Germany
9. to whom correspondence should be addressed:

   Breisacher Straße 115a

   D-79106 Freiburg (Germany)

Tel: +49 761 27080610

oliver.schilling@mol-med.uni-freiburg.de

Authors' email addresses and ORCIDs (same order as above):

melanie.foell@mol-med.uni-freiburg.de ORCID: 0000-0002-1887-7543, lennart.moritz@googlemail.com, thomas.wollmann@bioquant.uni-heidelberg.de ORCID: 0000-0002-4741-3844, maren.stillger@mol-med.uni-freiburg.de, vockert@stud.uni-heidelberg.de, martin.werner@uniklinik-freiburg.de, peter.bronsert@uniklinik-freiburg.de, k.rohr@dkfz-heidelberg.de, gruening@informatik.uni-freiburg.de ORCID: 0000-0002-3079-6586, oliver.schilling@mol-med.uni-freiburg.de. ORCID: 0000-0001-7678-7653

## Abstract:

Background:

Mass spectrometry imaging is increasingly used in biological and translational research as it has the ability to determine the spatial distribution of hundreds of analytes in a sample. Being at the interface of proteomics/metabolomics and imaging, the acquired data sets are large and complex and often analyzed with proprietary software or in-house scripts, which hinders reproducibility. Open source software solutions that enable reproducible data analysis often require programming skills and are therefore not accessible to many MSI researchers.

Findings:

We have integrated 18 dedicated mass spectrometry imaging tools into the Galaxy framework to allow accessible, reproducible, and transparent data analysis. Our tools are based on Cardinal, MALDIquant, and scikit-image and enable all major MSI analysis steps such as quality control, visualization, preprocessing, statistical analysis, and image co-registration. Further, we created hands-on training material for use cases in proteomics and metabolomics. To demonstrate the utility of our tools, we re-analyzed a publicly available N-

linked glycan imaging dataset. By providing the entire analysis history online, we highlight how the Galaxy framework fosters transparent and reproducible research.

Conclusion:

The Galaxy framework has emerged as a powerful analysis platform for the analysis of MSI data with ease of use and access together with high levels of reproducibility and transparency.


## Keywords:

Mass spectrometry imaging; MALDI imaging; spatially resolved mass spectrometry; proteomics; metabolomics; Galaxy; computational workflows; reproducibility


## Findings:

### Background:

Mass spectrometry imaging (MSI) is increasingly used for a broad range of biological and clinical applications as it allows the simultaneous measurement of hundreds of analytes and their spatial distribution. The versatility of MSI is based on its ability to measure many different kinds of molecules such as peptides, metabolites or chemical compounds in a large variety of samples such as cells, tissues, fingerprints or human made materials [1–5]. Depending on the sample, the analyte of interest and the application, different mass spectrometers are used [6]. The most common ionization sources are MALDI (Matrix Assisted Laser Desorption/Ionization), DESI (Desorption Electrospray Ionization) and SIMS (Secondary Ion Mass Spectrometry). Typical mass analyzers are time of flight (TOF) devices and ion traps.

Due to the variety of samples, analytes, and mass spectrometers, MSI is suitable for highly diverse use cases ranging from plant research, to (pre-)clinical, pharmacologic studies, and

forensic investigations [2, 7–9]. On the other hand, the variety of research fields hinders harmonization and standardization of MSI protocols. Recently efforts were started to develop optimized sample preparation protocols and show their reproducibility in multicenter studies [10–13]. In contrast, efforts to make data analysis standardized and reproducible are in its infancy.

Reproducibility of MSI data analyses is hindered by the common use of software with restricted access such as proprietary software, license requiring software, or unpublished in-house scripts [14]. Open source software has the potential to advance accessibility and reproducibility issues in data analysis but requires complete reporting of software versions and parameters, which is not yet routine in MSI [15–17].

At the same time, the introduction of the open standard file format imzML has opened new avenues to the community and an increasing number of open source software tools are emerging [18]. Yet, many of these tools necessitate steep learning curves, in some cases even requiring programming knowledge to make use of their full range of functions [19–23].

To overcome problems with accessibility of software and computing resources, standardization, and reproducibility, we developed MSI data analysis tools for the Galaxy framework that are based on the open source software suites Cardinal, MALDIquant, and scikit-image [20, 21, 24]. Galaxy is an open source computational platform for biomedical research that was developed to support researchers without programming skills with the analysis of large data sets, e.g. in the field of next generation sequencing. Galaxy is used by hundred thousands of researchers and provides thousands of different tools for many different scientific fields [25].

**Aims:**

With the present publication, we aim to raise awareness within the MSI community for the advantages being offered by the Galaxy framework with regard to standardized and

reproducible data analysis pipelines. Secondly, we present newly developed Galaxy tools and offer them to the MSI community through the graphical front-end and “drag-and-drop” workflows of the Galaxy framework. Thirdly, we apply the MSI Galaxy tools to a publicly available dataset to study N-glycan identity and distribution in murine kidney specimens in order to demonstrate usage of a Galaxy-based MSI analysis pipeline that facilitates standardization and reproducibility and is compatible with the principles of FAIR (findable, accessible, interoperable, and re-usable) data and MIAPE (minimum information about a proteomics experiment) [26, 27].

**The Galaxy framework for flexible and reproducible data analysis**

In essence, the Galaxy framework is characterized by four hallmarks: (1) usage of a graphical front-end that is web browser based, hence alleviating the need for advanced IT skills or the requirement to locally install and maintain software tools; (2) access to large-scale computational resources for academic users; (3) provenance tracking and full version control, including the ability to switch between software and tool version and to publish complete analysis, thus enabling full reproducibility; (4) access to a vast array of open-source tools with the ability of seamless passing data from one tool to another, thus generating added value by interoperability.

Multiple Galaxy servers on essentially every continent provide access to large computing resources, data storing capabilities, and hundreds of pre-installed tools for a broad range of data analysis applications through a web browser based graphical user interface [28–30]. Additionally, there are more than hundred public Galaxy servers available that offer more specific tools for niche application areas. For local usage, Galaxy can be installed on any computer ranging from private laptops to high computing clusters. So-called “containers” exist, which facilitate a fully functional one-click installation independent of the operating system. Hence, local Galaxy serves are easily deployed even in “private” network situations

in which these servers remain invisible and inaccessible to outside users. This ability empowers Galaxy for the analysis of sensitive and protected data, e.g. in a clinical setting.

In the Galaxy framework, data analysis information is stored alongside the results of each analysis step to ensure reproducibility and traceability of results. The information includes tool and software names and versions together with all parameters [31].

We propose that MSI research can greatly benefit from the possibility to privately or publicly share data analysis histories, workflows, and visualizations with collaboration partners or the entire scientific community, e.g. as online supplementary data for peer-reviewed publications. The latter step easily fulfills the criteria of the suggested MSI minimum reporting guidelines [6, 16].

The Galaxy framework is predestinated for the analysis of multi-omics studies as it facilitates the integration of software of different origin into one analysis [32, 33]. The possibility to seamlessly link tools of different origins has outstanding potential for MSI studies that often rely on different software platforms to analyze MSI data, additional MS/MS data (from liquid chromatography coupled tandem mass spectrometry), and (multimodal) imaging data. More than hundred tools for proteomic and metabolomics data analysis are readily available in Galaxy due to community driven efforts [34–38]. Increasing integration of MSI with other omics approaches such as genomics and transcriptomics is anticipated and the Galaxy framework offers a powerful and future-proof platform to tackle complex, interconnected data-driven experiments.

**The newly available MSI toolset in the Galaxy framework**

We have developed 18 Galaxy tools that are based on the commonly used open-source softwares Cardinal, MALDIquant, and scikit-image and enable all steps that commonly occur in MSI data analysis (Figure 1) [20, 21, 24]. In order to deeply integrate those tools into the Galaxy framework, we developed bioconda packages and biocontainers as well as a so called ‘wrapper’ for each tool [31, 39]. The MSI tools consist of R scripts that were developed

based on Cardinal and MALDIquant functionalities, extended for more analysis options and a consistent framework for input and output of metadata (Additional file 1). Cardinal and MALDIquant are well established R packages and are commonly used open source software for the analysis of MSI data [40–45]. Cardinal is under active development and provides a multitude of processing and analysis options for MSI data [46]. MALDIquant was originally developed for the analysis of classical MALDI-TOF data, but offers powerful preprocessing options that are applicable for the analysis of MSI data [44, 45]. The image processing tools that are part of the ROI annotation (co-registration) workflow are built from scratch using functionality from the scikit-image library. Scikit-image is an open-source image processing library for Python. All tools are deliberately built in a modular way to enable highly flexible analysis and to allow a multitude of additional functionalities by combining the MSI specific tools with already availably Galaxy tools.

Data formats and data handling:

We extended the Galaxy framework to support open and standardized MSI data files such as imzML, which is the default input format for the Galaxy MSI tools. Today, the major mass spectrometer vendors directly support the imzML standard and several tools exist to convert different file formats to imzML [47]. Data can be easily uploaded to Galaxy via a web browser or via a built-in file transfer protocol (FTP) functionality. Intermediate result files can be further processed in the interactive environment that supports R Studio and Jupyter or downloaded for additional analysis outside of Galaxy [48].

To facilitate the parallel analysis of multiple files, the Galaxy framework offers so-called “dataset collections”. Numerous files can be represented in a dataset collection allowing simultaneous analysis of all files while the effort for the user is similar as for single files. MSI meta data such as spectra annotations, calibrant m/z, and statistical results are stored as tab-separated values files, thus enabling processing by a plethora of tools both inside and

outside the Galaxy framework. All graphical results of the MSI tools are stored as concise vector graphic PDF reports with publication-quality images.

Quality control and visualization tools:

MSI Quality control: Quality control is an essential step in data analysis and should not only be used to judge the quality of the raw data but also to control processing steps such as smoothing, peak picking, and intensity normalization. Therefore, we have developed the 'MSI Qualitycontrol' tool that automatically generates a comprehensive pdf report with more than 30 different plots that enable a global view of all aspects of the MSI data including intensity distribution, m/z accuracy and segmentation maps (Figure 2). For example, poor quality spectra, such as low total ion current or low number of peaks can be directly spotted in the quality report and subsequently be removed by applying the 'MSI data exporter' and 'MSI filtering' tools.

MSI mz image: The 'MSI mz image' tool allows to automatically generate a publication-quality pdf file with distribution heat maps for all m/z features provided in a tab-separated values file. Contrast enhancement and smoothing options are available as well as the possibility to overlay several m/z features in one image (Figure 3 a, b).

MSI plot spectra: The 'MSI plot spectra' tool displays multiple single or average mass spectra in a pdf file. Overlay of multiple single or averaged mass spectra with different colors in one plot is also possible (Figure 3 c, d).

The Galaxy framework already offers various visualization options for tab-separated values files, including heatmaps, barplots, scatterplots, and histograms. This enables a quick visualization of the properties of tab-separated values files obtained during MSI analysis.

MSI file handling tools:

A large variety of tools that allows for filtering, sorting, and manipulating of tab-separated values files is already available in Galaxy and can be integrated into the MSI data analysis. Some dedicated tools for imzML file handling were newly integrated into the Galaxy framework.

MSI combine: The ‘MSI combine’ tool allows combining several imzML files into a merged dataset. This is especially important to enable direct visual but also statistical comparison of MSI data that derived from multiple files. With the ‘MSI combine tool’, individual MSI datasets are either placed next to each other in a coordinate system or can be shifted in x or y direction in a user defined way. The output of the tool contains a single file with the combined MSI data and an additional tab-separated values file with spectra annotations, i.e. each spectrum is annotated with its original file name (before combination) and, if applicable, with previously defined annotations such as diagnosis, disease type, and other clinical parameters.

MSI filtering: The ‘MSI filtering’ tool provides options to filter m/z features and pixel (spectra) of interest, either by applying manual ranges (minimum and maximum m/z, spatial area as defined by x / y coordinates) or by keeping only m/z features or coordinates of pixels that are provided in a tab-separated values file. Unwanted m/z features such as pre-defined contaminant features can be removed within a preselected m/z tolerance.

MSI data exporter: The ‘MSI data exporter’ can export the spectra, intensity and m/z data of an imzML file together with their summarized properties into tab-separated values files.

<u>Region of interest annotation tools:</u>

For supervised analysis, spatial regions of interest (ROI) can be defined. Those are commonly annotated on a photograph or histological image that shows the morphological features of the sample. We extended and developed six new Galaxy tools and combined them with existing tools into a workflow that enables co-registration of the real image

(photograph or histological image), ROIs, and the MSI image by alignment using an affine transformation [49]. The transformation is estimated by a least-squares method using landmarks from both real and MSI image which are annotated outside Galaxy, for example, using the GNU Image Manipulation Program (GIMP) (Figure 4) [50]. For more robust estimation of the transformation, Random sample consensus (RANSAC) is used on random subsets of landmark pairs [51].

The co-registration workflow includes six newly developed Galaxy tools. Scale Image: This tool can resize an image relative to the original image or using absolute dimensions with nearest neighbor, bilinear, or bicubic interpolation. Landmark registration: The landmark registration tool estimates the affine transformation between two sets of points using the random sample consensus (RANSAC) [51]. Overlay: This tool overlays two images, transforming one using a transformation matrix. The tool can be used to visually asses the performance of the registration. Coordinates of ROI: This tool extracts the indices of all pixels of a ROI from a binary image. Projective Transformation Points: This tool applies a transformation matrix to a set of points. Switch axis coordinates: The tool can be used to change the origin of a set of points in a coordinate system.

In the supporting information, we also provide automated workflows to convert annotation files from proprietary Bruker software (spotlist.txt and regions.xml) into annotation files that are compatible with the Galaxy MSI tools.

Preprocessing tools:

Preprocessing of raw MSI spectra is performed to reduce data size and to remove noise, inaccuracies and biases to improve downstream analysis. Crucial steps are peak picking to reduce file size and remove noise features, intensity normalization to make spectra within and between different samples comparable, as well as m/z recalibration to improve

comparability and identification of analytes. We have developed three dedicated MSI preprocessing tools that are based on a variety of preprocessing algorithms from both the Cardinal and MALDIquant package. An overview of all available preprocessing options is available in additional file 2.

MSI preprocessing: The ‘MSI preprocessing’ tool offers a multitude of algorithms that are useful to preprocess raw MSI data: intensity normalization to the total ion current (TIC), baseline removal, smoothing, peak picking, peak alignment, peak filtering, intensity transformation, binning and resampling.

MALDIquant preprocessing and MALDIquant peak detection: Both MALDIquant tools offer a multitude of preprocessing algorithms that complement those of the cardinal based MSI preprocessing tool such as m/z re-calibration, peak picking on average mass spectra and picking of monoisotopes.

<u>Statistical analysis tools:</u>

A multitude of statistical analysis options for tab-separated values files is already available in Galaxy, the most MSI relevant tools are from the Workflow4metabolomics project and consist of unsupervised and supervised statistical analysis tools [52]. For specific purposes of spatially resolved MSI data analysis, we have integrated Cardinal’s powerful spatially aware statistical analysis options into the Galaxy framework.

MSI segmentation: The ‘MSI segmentation’ tool enables spatially aware unsupervised statistical analysis with principal component analysis, spatially aware k-means clustering and spatial shrunken centroids [53, 54].

MSI classification: The ‘MSI classification’ tool offers three options for spatially aware supervised statistical analysis: partial least square (discriminant analysis), orthogonal partial least squares (discriminant analysis), and spatial shrunken centroids [53].

Analyte identification tools:

m/z determination on its own often remains insufficient to identify analytes. Compound fragmentation and tandem mass spectrometry are typically employed for compound identification by mass spectrometry. In MSI, the required local confinement of the mass spectrometry analysis severely limits the compound amounts that are available for fragmentation. Hence, direct on-target fragmentation is rarely employed in MSI. A common practice for compound identification includes a combinatorial approach in which LC-MS/MS data is used to identify the analytes while MSI analyses their spatial distribution. This approach requires assigning putative analyte information to m/z values within a given accuracy range.

Join two files on a column allowing a small difference: This newly developed tool allows for the matching of numeric columns of two tab-separated values files on the smallest distance that can be absolute or in ppm. This tool can be used to identify the m/z features of a tab-separated values files by matching them to already identified m/z features of another tab-separated values file (e.g. from a database or from an analysis workflow).

Community efforts such as Galaxy-M, Galaxy-P, Phenomenal, and Workflow4Metabolomics have led to a multitude of metabolomics and proteomics analysis tools that are available in Galaxy today [34–38]. These tools allow analyzing additional tandem mass spectrometry data that is often acquired to aid identification of MSI m/z features. Databases to which the results can be matched, such as uniprot and lipidmaps, are directly available in Galaxy [55, 56]. The highly interdisciplinary and modular data analysis options in Galaxy render it a very powerful platform for MSI data analyses that are part of a multi-omics study.

**Accessibility & training**

All described tools are easily accessible and usable via the European Galaxy server [29]. Furthermore, all tools are deposited in the Galaxy Toolshed from where they can be easily installed into any other Galaxy instance (Additional file 3) [57]. We have developed bioconda packages and biocontainers that allow for version control and automated installation of all tool dependencies – those packages are also useful outside Galaxy to enhance reproducibility [31, 39]. For researchers that do not want to use publicly available Galaxy servers, we provide a pre-built Docker image that is easy to install independent of the operating system.

For a swift introduction into the analysis of MSI data in Galaxy, we have developed training material for metabolomics and proteomic use cases and deposited it to the central repository of the Galaxy Training Network [58, 59]. The training materials consist of a comprehensive collection of small example datasets, step-by-step explanations and workflows that enable any interested researcher in following the training and understanding it through active participation.

The first training explains data upload in Galaxy and describes the quality control of mouse kidney tissue section in which peptides were imaged with an old MALDI-TOF [60]. The dataset contains peptide calibrants that allow the control of the digestion efficiency and m/z accuracy. Export of MSI data into tab-separated values files and further filtering of those files is explained as well.

The second training explains the examination of the spatial distribution of volatile organic compounds in a chilli section. The training roughly follows the corresponding publication and explains how average mass spectra are plotted and only the relevant m/z range is kept, as well as how to automatically generate many m/z distribution maps and overlay several m/z feature maps [19].

The third training determines and identifies N-linked glycans in mouse kidney tissue sections with MALDI-TOF and additional LC-MS/MS data analysis [61, 62]. The training covers combining datasets, preprocessing as well as unsupervised and supervised statistical

analysis to find potential N-linked glycans that have different abundances in the PNGase F treated kidney section compared to the kidney section that was treated with buffer only. The training further covers identification of the potential N-linked glycans by matching their m/z values to a list of N-linked glycan m/z that were identified by LC-MS/MS. The full dataset is used as a case study in the following section.

**Case study**

To exemplify the utility of our MSI tools we re-analyzed the N-glycan dataset that was recently made available by Gustafsson et al. via the PRIDE repository with accession PXD009808 [62, 63]. The aim of the study was to demonstrate that their automated sample preparation method for MALDI imaging of N-linked glycans successfully works on formalin-fixed paraffin-embedded (FFPE) murine kidney tissue [61]. PNGase F was printed on two FFPE murine kidney sections to release N-linked glycans from proteins while in a third section one part of the kidney was covered with N-glycan calibrants and another part with buffer to serve as a control. The tissues were measured with a MALDI-TOF/TOF mass spectrometer and a spatial resolution of 100 µm that leads to oversampling of the 250 µm PNGase F array [61]. We downloaded all four imzML files (two treated kidneys, control and calibrants) from PRIDE and uploaded them with the composite upload function into Galaxy. To obtain an overview of the files we used the ‘MSI Qualitycontrol’ tool. We resampled the m/z axis, combined all files and run again the ‘MSI Qualitycontrol’ tool to directly compare the four subfiles (additional file 4). Next, we performed TIC normalization, smoothing and baseline removal by applying Cardinal algorithms [21]. Spectra were aligned to the stable peaks that are present in at least 80 % of all spectra [64]. Spectra, in which less than two stable peaks could be aligned, were removed. This affected mainly spectra from the control file. Peak picking, detection of monoisotopic peaks and binning was performed on the average spectra of each subfile [64]. The obtained m/z features were extracted with Cardinal’s ‘peaks’ algorithm from the normalized, smoothed, baseline removed and aligned

file. Next, principal component analysis with four components was performed (Figure 5) [21]. To find potential N-linked glycans, the two treated tissues were compared to the control tissue with the supervised spatial shrunken centroids algorithm [53]. Spatial shrunken centroids is a multivariate classification method that was specifically developed to account for the spatial structure of the data (Figure 6a) [53]. The supervised analysis provided us with 28 m/z features that discriminated between the two PNGase F treated kidneys and the control kidney with a spatial shrunken centroids p-value $< 0.05$ and higher abundance in the treated kidneys. Mapping those features to N-glycans reported in the original publication (Gustafsson et al., Supplementary Table S2) revealed the identity of 16 N-glycans with an average m/z error of 49 ppm (Table 1). Fifteen of those N-glycans match to the findings of the original publication. Whilst our workflow did not identify the reported N-glycan at 1647.635 m/z, an additional N-glycan at 1542.62 m/z was found. The intensity distribution for four N-glycans on the TIC normalized dataset is depicted in Figure 6b-e and three of them are overlayed in Figure 6f.

PLACEHOLDER FOR TABLE 1 (larger than DinA4 portrait format)

The complete analysis was performed in the European Galaxy instance with MSI tools based on Cardinal version 1.12.1 and MALDIquant 1.18 [21, 29]. Despite having used different algorithms for preprocessing and statistical analysis, we reached similar findings as compared to Gustafsson. The reproducibility of the results shows the capacity of our pipeline. To enable full “methods reproducibility” we provide the analysis history and workflow in this publication as supporting information. Those can be easily published on the Galaxy platform and provide more information than requested by the minimum reporting guidelines MSI MIAPE (Minimum Information About a Proteomics Experiment) and MIAMSIE (Minimum Information About a Mass Spectrometry Imaging Experiment) [6, 16]. The Galaxy software itself but also the shared histories and workflows fulfill the FAIR principles that stand for findability, accessibility, interoperability, and reusability [27].

**Summary**

With the integration of the MSI data analysis toolset, we have incorporated an accessible and reproducible data analysis platform for MSI data in the Galaxy framework. Our MSI tools complement the multitude of already available Galaxy tools for proteomics and metabolomics that are maintained by Galaxy-M, Galaxy-P, Phenomal and Workflow4Metabolomics [34–38]. We are in close contact with those communities and would like to encourage developers of the MSI community to join forces and make their tools available in the Galaxy framework. We currently focused on reproducible and accessible data analysis, but we are planning to integrate interactive visualizations, more support for very large files and more tools for specific use cases into the Galaxy framework. Lastly, we would like to invite the MSI community to use the advantages of the Galaxy framework to advance MSI data analysis.

## Availability of supporting source code and requirements

Project name: Mass spectrometry imaging workbench in Galaxy

RRID number: SCR_017410

Project homepage: https://github.com/galaxyproteomics/tools-galaxyp and https://github.com/BMCV/galaxy-image-analysis

Galaxy Toolshed: https://toolshed.g2.bx.psu.edu/

Operating system(s): Unix (Platform independent with Docker)

Training repository: https://galaxyproject.github.io/training-material/ ‘mass spectrometry imaging’ tutorials can be found in the sections ‘metabolomics’ and ‘proteomics’.

Docker image: https://github.com/foellmelanie/docker-galaxy-msi

License: MIT

## Additional files

Additional file 1: Overview of R-functions in the MSI tools. For each Galaxy MSI tool the R-functions that do not belong to the basic R-package are listed.

Additional file 2: Overview of available preprocessing options.

Additional file 3: Collection of direct links to the toolshed location for each tool.

Additional file 4: Exemplary quality control plots for the combined N-glycan imaging file

## Availability of supporting data

Galaxy workflow to convert Bruker ROI.xml files: https://usegalaxy.eu/u/melanie-foell/w/msi-workflow-bruker-xml-conversion-to-tabular-file

Galaxy workflow to convert Bruker spotlists: https://usegalaxy.eu/u/melanie-foell/w/bruker-spotlist-conversion-to-tabular-file

Galaxy workflow co-registration: https://usegalaxy.eu/u/melanie-foell/w/co-registration-of-msi-image-and-real-image-with-landmarks

Galaxy workflow N-linked glycans re-analysis: https://usegalaxy.eu/u/melanie-foell/w/msi-workflow-complete-n-glycan-analysis

Galaxy history N-linked glycans re-analysis: https://usegalaxy.eu:/u/melanie-foell/h/re-analysis-of-pride-dataset-pxd009808---maldi-imaging-of-n-linked-glycans-in-murine-kidney-specimens

Archival copies of the code and workflows are available from the *GigaScience* GigaDB repository [65].

## List of abbreviations

DESI: Desorption Electrospray Ionization; MALDI: Matrix Assisted Laser Desorption/Ionization; FAIR: findable, accessible, interoperable, and re-usable; FFPE: formalin-fixed paraffin-embedded; LC-MS/MS: liquid chromatography tandem mass spectrometry; MIAMSIE: Minimum Information About a Mass Spectrometry Imaging Experiment; MIAPE: minimum information about a proteomics experiment; MSI: Mass spectrometry imaging; PRIDE: proteomics identifications; ROI: Region of interest; SIMS: Secondary Ion Mass Spectrometry; TOF: Time of flight

## Competing interests

The authors declare that they have no competing interests.

## Funding

OS acknowledges support by the German Research Council (DFG, GR 1748/6-1, SCHI 871/8-1, SCHI 871/9-1, SCHI 871/11-1, SCHI 871/12-1, INST 39/900-1, and SFB850-Project Z1 (INST 39/766-3), RO-5694/1-1), the German-Israel Foundation (Grant No. I-1444-201.2/2017), and the European Research Council (780730, ProteaseNter, ERC-2017-PoC).

B.A.G. is supported by the German Federal Ministry of Education and Research (031L0101C de.NBI-epi) and the European Open Science Cloud (EOSC-Life) (Grant No. 824087).

K.R. acknowledges support of the Federal Ministry of Education and Research (de.NBI, CancerTelSys) and the German Research Foundation (SFB 1129, RTG 1653).

The article processing charge was funded by the German Research Foundation (DFG) and the University of Freiburg in the funding programme Open Access Publishing.

## Authors' contributions

M.C.F. developed the MSI Galaxy tool wrappers, the training material and the case study. L.M. acquired data for the training material, tested MSI tools and training material and

provided useful feedback. T.W. developed the Galaxy tools and workflow for co-registration and contributed to build the Galaxy tool wrappers. M.N.S. tested MSI tools, co-registration tools and training material and provided useful feedback. N.V. built the Galaxy tool wrappers for the co-registration tools and tested them. M.W., P.B., K.R., B.A.G., O.S. contributed to the conceptualization, methodology, and funding acquisition. B.A.G. integrated the MSI file formats into Galaxy, contributed to build the training material and tool wrappers and integrated all tool wrappers into Galaxy. O.S. and M.C.F. wrote the manuscript. All authors critically read and approved the manuscript's contents.

## Acknowledgement

We thank the European Galaxy Instance for bioinformatics support (https://usegalaxy.eu/) and the Galaxy community for critically reviewing tools and training material.

## References

1. Yang B, Patterson NH, Tsui T, Caprioli RM, Norris JL. Single-Cell Mass Spectrometry Reveals Changes in Lipid and Metabolite Expression in RAW 264.7 Cells upon Lipopolysaccharide Stimulation. J Am Soc Mass Spectrom. 2018;29:1012–20. doi:10.1007/s13361-018-1899-9.

2. Bhandari DR, Wang Q, Friedt W, Spengler B, Gottwald S, Römpp A. High resolution mass spectrometry imaging of plant tissues: towards a plant metabolite atlas. Analyst. 2015;140:7696–709. doi:10.1039/C5AN01065A.

3. Bradshaw R, Bleay S, Clench MR, Francese S. Direct detection of blood in fingermarks by MALDI MS profiling and Imaging. Sci Justice. 2014;54:110–7. doi:10.1016/j.scijus.2013.12.004.

4. Correa DN, Zacca JJ, Rocha WF de C, Borges R, de Souza W, Augusti R, et al. Anti-theft device staining on banknotes detected by mass spectrometry imaging. Forensic Sci Int. 2016;260:22–6. doi:10.1016/j.forsciint.2015.09.017.

5. Kramell AE, García-Altares M, Pötsch M, Kluge R, Rother A, Hause G, et al. Mapping Natural Dyes in Archeological Textiles by Imaging Mass Spectrometry. Sci Rep. 2019;9:2331. doi:10.1038/s41598-019-38706-4.

6. McDonnell LA, Römpp A, Balluff B, Heeren RMA, Albar JP, Andrén PE, et al. Discussion point: reporting guidelines for mass spectrometry imaging. Anal Bioanal Chem. 2015;407:2035–45.

7. Vaysse PM, Heeren RMA, Porta T, Balluff B. Mass spectrometry imaging for clinical research-latest developments, applications, and current limitations. Analyst. 2017;142:2690–712.

8. Karlsson O, Hanrieder J. Imaging mass spectrometry in drug development and toxicology. Arch Toxicol. 2017;91:2283–94. doi:10.1007/s00204-016-1905-6.

9. Hoffmann WD, Jackson GP. Forensic Mass Spectrometry. Annu Rev Anal Chem. 2015;8:419–40. doi:10.1146/annurev-anchem-071114-040335.

10. Römpp A, Both JP, Brunelle A, Heeren RMA, Laprévote O, Prideaux B, et al. Mass spectrometry imaging of biological tissue: an approach for multicenter studies. Anal Bioanal Chem. 2015;407:2329–35.

11. Buck A, Heijs B, Beine B, Schepers J, Cassese A, Heeren RMA, et al. Round robin study of formalin-fixed paraffin-embedded tissues in mass spectrometry imaging. Anal Bioanal Chem. 2018;410:5969–80.

12. Porcari AM, Zhang J, Garza KY, Rodrigues-Peres RM, Lin JQ, Young JH, et al. Multicenter Study Using Desorption-Electrospray-Ionization-Mass-Spectrometry Imaging for Breast-Cancer Diagnosis. Anal Chem. 2018;90:11324–32.

13. Ly A, Longuespée R, Casadonte R, Wandernoth P, Schwamborn K, Bollwein C, et al. Site-to-Site Reproducibility and Spatial Resolution in MALDI–MSI of Peptides from Formalin-Fixed Paraffin-Embedded Samples. Proteomics - Clin Appl. 2019;13:1800029.

doi:10.1002/prca.201800029.

14. Gessel MM, Norris JL, Caprioli RM. MALDI imaging mass spectrometry: Spatial molecular analysis to enable a new age of discovery. J Proteomics. 2014;107:71–82. doi:10.1016/j.jprot.2014.03.021.

15. Grüning B, Chilton J, Köster J, Dale R, Soranzo N, van den Beek M, et al. Practical Computational Reproducibility in the Life Sciences. Cell Syst. 2018;6:631–5. doi:10.1016/j.cels.2018.03.014.

16. Gustafsson OJR, Winderbaum LJ, Condina MR, Boughton BA, Hamilton BR, Undheim EAB, et al. Balancing sufficiency and impact in reporting standards for mass spectrometry imaging experiments. Gigascience. 2018;7:1–13. doi:10.1093/gigascience/giy102.

17. Gruening B, Sallou O, Moreno P, da Veiga Leprevost F, Ménager H, Søndergaard D, et al. Recommendations for the packaging and containerizing of bioinformatics software. F1000Research. 2018;7:742. doi:10.12688/f1000research.15140.1.

18. Schramm T, Hester A, Klinkert I, Both JP, Heeren RMA, Brunelle A, et al. ImzML - A common data format for the flexible exchange and processing of mass spectrometry imaging data. J Proteomics. 2012;75:5106–10. doi:10.1016/j.jprot.2012.07.026.

19. Gamboa-Becerra R, Ramírez-Chávez E, Molina-Torres J, Winkler R. MSI.R scripts reveal volatile and semi-volatile features in low-temperature plasma mass spectrometry imaging (LTP-MSI) of chilli (Capsicum annuum). Anal Bioanal Chem. 2015;407:5673–84. doi:10.1007/s00216-015-8744-9.

20. Gibb S, Strimmer K. Maldiquant: A versatile R package for the analysis of mass spectrometry data. Bioinformatics. 2012;28:2270–1.

21. Bemis KD, Harry A, Eberlin LS, Ferreira C, Van De Ven SM, Mallick P, et al. Cardinal: An R package for statistical analysis of mass spectrometry-based imaging experiments. Bioinformatics. 2015;31:2418–20. doi:10.1093/bioinformatics/btv146.

22. Veselkov K, Sleeman J, Claude E, Vissers JPC, Galea D, Mroz A, et al. BASIS: High-performance bioinformatics platform for processing of large-scale mass spectrometry imaging data in chemically augmented histology. Sci Rep. 2018;8:1–11. doi:10.1038/s41598-018-22499-z.

23. Ràfols P, Torres S, Ramírez N, Del Castillo E, Yanes O, Brezmes J, et al. RMSI: An R package for MS imaging data handling and visualization. Bioinformatics. 2017;33:2427–8. doi:10.1093/bioinformatics/btx182.

24. van der Walt S, Schönberger JL, Nunez-Iglesias J, Boulogne F, Warner JD, Yager N, et al. scikit-image: image processing in Python. PeerJ. 2014;2:e453. doi:10.7717/peerj.453.

25. Guerler A, Baker D, Clements D, Afgan E, Taylor J, Batut B, et al. The Galaxy platform for accessible, reproducible and collaborative biomedical analyses: 2018 update. Nucleic Acids Res. 2018;46:W537–44. doi:10.1093/nar/gky379.

26. Taylor CF, Paton NW, Lilley KS, Binz P, Julian RK, Jones AR, et al. The minimum information about a proteomics experiment (MIAPE). Nat Biotechnol. 2007;25:887–93. doi:10.1038/nbt1329.

27. Wilkinson MD. Comment: The fair guiding principles for scientific data management and stewardship. Sci Data. 2016;:1–9.

28. Main Galaxy Instance. https://usegalaxy.org/. Accessed 2 Apr 2019.

29. European Galaxy Instance. https://usegalaxy.eu/. Accessed 9 Mar 2019.

30. Australian Galaxy Instance. https://usegalaxy.org.au/. Accessed 2 Apr 2019.

31. Grüning B, Dale R, Sjödin A, Chapman BA, Rowe J, Tomkins-Tinch CH, et al. Bioconda: sustainable and comprehensive software distribution for the life sciences. Nat Methods. 2018;15:475–6. doi:10.1038/s41592-018-0046-7.

32. Boekel J, Chilton JM, Cooke IR, Horvatovich PL, Jagtap PD, Käll L, et al. Multi-omic data analysis using Galaxy. Nat Biotechnol. 2015;33:137–9. doi:10.1038/nbt.3134.

33. Heydarian M. Prediction of Gene Activity in Early B Cell Development Based on an Integrative Multi-Omics Analysis. J Proteomics Bioinform. 2014;7. doi:10.4172/jpb.1000302.

34. Davidson RL, Weber RJM, Liu H, Sharma-Oates A, Viant MR. Galaxy-M: A Galaxy workflow for processing and analyzing direct infusion and liquid chromatography mass spectrometry-based metabolomics data. Gigascience. 2016;5. doi:10.1186/s13742-016-0115-8.

35. Jagtap PD, Johnson JE, Onsongo G, Sadler FW, Murray K, Wang Y, et al. Flexible and accessible workflows for improved proteogenomic analysis using the galaxy framework. J Proteome Res. 2014;13:5898–908. doi:10.1021/pr500812t.

36. Peters K, Bradbury J, Bergmann S, Capuccini M, Cascante M, de Atauri P, et al. PhenoMeNal: processing and analysis of metabolomics data in the cloud. Gigascience. 2019;8:1–12. doi:10.1093/gigascience/giy149.

37. Guitton Y, Tremblay-Franco M, Le Corguillé G, Martin JF, Pétéra M, Roger-Mele P, et al. Create, run, share, publish, and reference your LC–MS, FIA–MS, GC–MS, and NMR data analysis workflows with the Workflow4Metabolomics 3.0 Galaxy online infrastructure for metabolomics. Int J Biochem Cell Biol. 2017;93 January:89–101. doi:10.1016/j.biocel.2017.07.002.

38. Galaxy-P Github repository. https://github.com/galaxyproteomics/tools-galaxyp. Accessed 2 Apr 2019.

39. da Veiga Leprevost F, Grüning BA, Alves Aflitos S, Röst HL, Uszkoreit J, Barsnes H, et al. BioContainers: an open-source and community-driven framework for software standardization. Bioinformatics. 2017;33:2580–2.

40. van de Ven SMWY, Bemis KD, Lau K, Adusumilli R, Kota U, Stolowitz M, et al. Protein biomarkers on tissue as imaged via MALDI mass spectrometry: A systematic approach to study the limits of detection. Proteomics. 2016;16:1660–9.

41. Erich K, Sammour DA, Marx A, Hopf C. Scores for standardization of on-tissue digestion of formalin-fixed paraffin-embedded tissue in MALDI-MS imaging. Biochim Biophys Acta - Proteins Proteomics. 2017;1865:907–15. doi:10.1016/j.bbapap.2016.08.020.

42. Patterson NH, Tuck M, Van De Plas R, Caprioli RM. Advanced Registration and Analysis of MALDI Imaging Mass Spectrometry Measurements through Autofluorescence Microscopy. Anal Chem. 2018;90:12395–403.

43. Patterson NH, Alabdulkarim B, Lazaris A, Thomas A, Marcinkiewicz MM, Gao ZH, et al. Assessment of pathological response to therapy using lipid mass spectrometry imaging. Sci Rep. 2016;6 June:1–13. doi:10.1038/srep36814.

44. Abu Sammour D, Marsching C, Geisel A, Erich K, Schulz S, Ramallo Guevara C, et al. Quantitative Mass Spectrometry Imaging Reveals Mutation Status-independent Lack of Imatinib in Liver Metastases of Gastrointestinal Stromal Tumors. Sci Rep. 2019;9:10698. doi:10.1038/s41598-019-47089-5.

45. Inglese P, Correia G, Pruski P, Glen RC, Takats Z. Colocalization Features for Classification of Tumors Using Desorption Electrospray Ionization Mass Spectrometry Imaging. Anal Chem. 2019;91:6530–40.

46. Cardinal Github Repository. https://github.com/kuwisdelu/Cardinal. Accessed 15 Aug 2019.

47. Mass Spectrometry Imaging Society: Software tools. https://ms-imaging.org/wp/imzml/software-tools/. Accessed 9 Mar 2019.

48. Grüning BA, Rasche E, Rebolledo-Jaramillo B, Eberhard C, Houwaart T, Chilton J, et al. Jupyter and Galaxy: Easing entry barriers into complex data analyses for biomedical researchers. PLoS Comput Biol. 2017;13:e1005425.

49. Wollmann T, Erfle H, Eils R, Rohr K, Gunkel M. Workflows for microscopy image analysis and cellular phenotyping. J Biotechnol. 2017;261 July:70–5.

50. GNU Image Manipulation Program (GIMP). https://www.gimp.org/. Accessed 2 Apr 2019.

51. Fischler MA, Bolles RC. Random sample consensus: a paradigm for model fitting with applications to image analysis and automated cartography. Commun ACM. 1981;24:381–95. doi:10.1145/358669.358692.

52. Giacomoni F, Le Corguillé G, Monsoor M, Landi M, Pericard P, Pétéra M, et al. Workflow4Metabolomics: A collaborative research infrastructure for computational metabolomics. Bioinformatics. 2015;31:1493–5.

53. Bemis KD, Harry A, Eberlin LS, Ferreira CR, van de Ven SM, Mallick P, et al. Probabilistic Segmentation of Mass Spectrometry (MS) Images Helps Select Important Ions and Characterize Confidence in the Resulting Segments. Mol Cell Proteomics. 2016;15:1761–72. doi:10.1074/mcp.O115.053918.

54. Alexandrov T, Kobarg JH. Efficient spatial segmentation of large imaging mass spectrometry datasets with spatially aware clustering. Bioinformatics. 2011;27:230–8.

55. Bateman A. UniProt: A worldwide hub of protein knowledge. Nucleic Acids Res. 2019;47:D506–15. doi:10.1093/nar/gky1049.

56. Sud M, Fahy E, Cotter D, Brown A, Dennis EA, Glass CK, et al. LMSD: LIPID MAPS structure database. Nucleic Acids Res. 2007;35 SUPPL. 1:D527–32. doi:10.1093/nar/gkl838.

57. Blankenberg D, Von Kuster G, Bouvier E, Baker D, Afgan E, Stoler N, et al. Dissemination of scientific software with Galaxy ToolShed. Genome Biol. 2014;15:403. doi:10.1186/gb4161.

58. Batut B, Hiltemann S, Bagnacani A, Baker D, Bhardwaj V, Blank C, et al. Community-Driven Data Analysis Training for Biology. Cell Syst. 2018;6:752–758.e1. doi:10.1016/j.cels.2018.05.012.

59. Galaxy Training Network. https://galaxyproject.github.io/training-material/. Accessed 9 Mar 2019.

60. Moritz L, Föll M, Schilling O. MALDI imaging of mouse kidney peptides - test dataset on Zenodo. 2018. doi:10.5281/zenodo.1560645.

61. Gustafsson OJR, Briggs MT, Condina MR, Winderbaum LJ, Pelzing M, McColl SR, et al. MALDI imaging mass spectrometry of N-linked glycans on formalin-fixed paraffin-embedded murine kidney. Anal Bioanal Chem. 2015;407:2127–39. doi:10.1007/s00216-014-8293-7.

62. Gustafsson OJR, Briggs MT, Condina MR, Winderbaum LJ, Pelzing M, McColl SR, et al. Raw N-glycan mass spectrometry imaging data on formalin-fixed mouse kidney. Data Br. 2018;21:185–8. doi:10.1016/j.dib.2018.08.186.

63. Vizcaíno JA, Csordas A, Del-Toro N, Dianes JA, Griss J, Lavidas I, et al. 2016 update of the PRIDE database and its related tools. Nucleic Acids Res. 2016;44:D447–56. doi:10.1093/nar/gkv1145.

64. Gibb S, Strimmer K. Mass Spectrometry Analysis Using MALDIquant. In: Statistical Analysis of Proteomics, Metabolomics, and Lipidomics Data Using Mass Spectrometry. Cham: Springer International Publishing; 2017. p. 101–24. doi:10.1007/978-3-319-45809-0_6.

65. Foell MC; Moritz L; Wollmann T; Stillger M; Vockert N; Werner M; Bronsert P; Rohr K; Gruening B; Schilling O (2019): Supporting data for "Accessible and reproducible mass spectrometry imaging data analysis in Galaxy" GigaScience Database.

http://dx.doi.org/10.5524/100665

66. Batut B, Hiltemann S, Bagnacani A, Baker D, Bhardwaj V, Blank C, et al. Community-Driven Data Analysis Training for Biology. Cell Syst. 2018;6:752–758.e1. doi:10.1016/j.cels.2018.05.012.

# Figures and larger than DinA4 Tables

**Table 1: N-linked Glycans identified in the re-analysis.**

| mz | centers | tstatistics | adj.p.values | M+Na+ | composition |
|---|---|---|---|---|---|
| **1257.47424** | 38.24 | 51.97 | 0 | 1257.41 | $(Hex)_2+(Man)_3(GlcNAc)_2$ |
| **1743.68713** | 32.11 | 48.56 | 0 | 1743.57 | $(Hex)_5+(Man)_3(GlcNAc)_2$ |
| **1419.55334** | 40.68 | 48.2 | 0 | 1419.47 | $(Hex)_3+(Man)_3(GlcNAc)_2$ |
| **1905.68713** | 48.61 | 44.78 | 0 | 1905.63 | $(Hex)_6+(Man)_3(GlcNAc)_2$ |
| **2304.91211** | 43.53 | 42.36 | 0 | 2304.83 | $(Hex)_2(HexNAc)_3(Deoxyhexose)_3+(Man)_3(GlcNAc$ |
| **1850.71216** | 25.3 | 42.01 | 0 | 1850.65 | $(Hex)_1(HexNAc)_3(Deoxyhexose)_1+(Man)_3(GlcNAc$ |
| **1581.62573** | 18.07 | 40.64 | 0 | 1581.53 | $(Hex)_4+(Man)_3(GlcNAc)_2$ |
| **1809.72461** | 10.81 | 38.15 | 0 | 1809.63 | $(Hex)_2(HexNAc)_2(Deoxyhexose)_1+(Man)_3(GlcNAc$ |
| **2158.88721** | 14.77 | 38.03 | 0 | 2158.77 | $(Hex)_2(HexNAc)_3(Deoxyhexose)_2+(Man)_3(GlcNAc$ |
| **1663.66638** | 10.27 | 32.26 | 0 | 1663.57 | $(Hex)_2(HexNAc)_2+(Man)_3(GlcNAc)_2$ |
| **1688.71509** | 8.68 | 28.29 | 0 | 1688.61 | $(HexNAc)_3(Deoxyhexose)_1+(Man)_3(GlcNAc)_2$ |
| **1485.62378** | 8.67 | 26.89 | 0 | 1485.53 | $(HexNAc)_2(Deoxyhexose)_1+(Man)_3(GlcNAc)_2$ |
| **2012.78394** | 7.3 | 26.72 | 0 | 2012.71 | $(Hex)_2(HexNAc)_3(Deoxyhexose)_1+(Man)_3(GlcNAc$ |
| **2816.11206** | 6.92 | 26.35 | 0 | 2816.01 | $(Hex)_3(HexNAc)_4(Deoxyhexose)_1+(Man)_3(GlcNAc$ |
| **2067.75903** | 5.69 | 14.52 | 0 | 2067.67 | $(Hex)_7+(Man)_3(GlcNAc)_2$ |
| **1542.61902** | 5.59 | 8.08 | 0 | 1542.55 | $(HexNAc)_3+(Man)_3(GlcNAc)_2$ |

We could identify 16 N-linked glycans by matching the m/z features of the MSI data (column 1) to the identified m/z features of the LC-MS/MS experiment (column 5). We allowed a maximum tolerance of 300 ppm and multiple matches. Only single matches occurred with an average m/z error of 46 ppm (column 6).

**Figure 1: Typical MSI data analysis steps and newly developed Galaxy tools.**

Typical MSI data analysis step include Quality control, file handling, preprocessing, ROI annotation, supervised and unsupervised statistical analysis, visualizations and identification of features. Due to the variety of MSI applications, tools of all or only a few of these categories are used and the order of usage is highly flexible. To serve a broad range of data analysis tasks, we provide 18 tools that cover all common data analysis procedures and can be arbitrarily connected to allow customized analysis. Dark blue: MSI data analysis steps, pale blue: newly developed Galaxy tools.

**Figure 2: Exemplary quality control plots.**

The Quality control report contains more than 30 different plots, 5 of them are shown here. They derive from the peptide imaging in mouse kidney training that we provide in the Galaxy training network [66] a) Control of tryptic digestion with a bombesin spot next to the tissue. The log 2 fold change of the cleaved vs. uncleaved bombesin peptide shows the digestion efficiency in the spot. b) Total ion current (TIC) in each pixel c) m/z accuracy for the internal calibrant angiotensin I in each pixel d) average mass spectrum showing the angiotensin I peak and its first isotope together with vertical lines indicating the theoretical m/z of angiotensin, the most abundant m/z in the chosen window and the closest m/z value in the file in green e) TIC for each spectrum.

**Figure 3: Visualization of heat maps and mass spectra.**

Visualizations of heat maps, overlay image and mass spectra plots of a chilli section that is part of the chilli training that we provide in the Galaxy training network [19, 58]. a) Heat map of the m/z feature 306.6 that corresponds to capsaicin. b) Overlay of the m/z features 306.6, 62.2 and 84.2 that show different distribution in the chilli section. c) Average mass spectrum for the complete chilli dataset. d) Overlay of single mass spectra that belong to different chilli compartments: green: pericarp, light blue: placenta, purple: seeds, red: average mass spectra of all other chilli spectra.

**Figure 4: Co-registration strategy using affine transformation and landmarks.**

Co-registration of an MSI image with a histological H&E image from a mouse kidney via affine transformation. The affine transformation is estimated from the landmarks (red dots) that the user has to provide. Landmarks can be either characteristic marks of the sample or marks applied outside the tissue e.g. with a xylene resistant pen (black crosses). The transformation matrix, estimated by the affine transformation estimation tool, can be used to generate an overlay of both images (right image) for visual inspection. Moreover, the matrix can be used to transfer the annotated regions of interest in the H&E image to the MSI coordinates (not shown here).

**Figure 5: Results from the unsupervised statistical analysis of N-linked glycans in murine kidney tissues.**

Principal component analysis of treated, control and calibrant files. a) Overlay of all principal component scores. b-d) Principal components 1,3 and 4 that discriminate treated and control

tissue or different kidney compartments. I = treated kidney1, II = treated kidney2, III = control kidney, IV = calibrants.

**Figure 6: Results from the supervised statistical analysis of N-linked glycans in murine kidney tissues.**

The supervised spatial shrunken centroids method was used to determine m/z features that are more abundant in the treated than in the control tissue specimen. a) Spatial shrunken centroids class prediction for all spectra. b-e) Intensity distribution images for four identified N-linked glycans $(Hex)_2(HexNAc)_2(Deoxyhexose)_1$+$(Man)_3(GlcNAc)_2$ (m/z 1663.6), $(Hex)_6$+$(Man)_3(GlcNAc)_2$ (m/z 1905.7), $(Hex)_2(HexNAc)_3(Deoxyhexose)_3$+$(Man)_3(GlcNAc)_2$ (m/z 2304.9) and $(Hex)_3(HexNAc)_4(Deoxyhexose)_1$+$(Man)_3(GlcNAc)_2$(m/z 2816.0). f) Overlay of three N-linked glycans with different distribution in the kidney. The ion distribution images and the overlay image were generated with contrast enhancement by suppression on TIC normalized data. I = treated kidney1, II = treated kidney2, III = control kidney, IV = calibrants.

Figure

Click here to access/download;Figure;Figure1.png

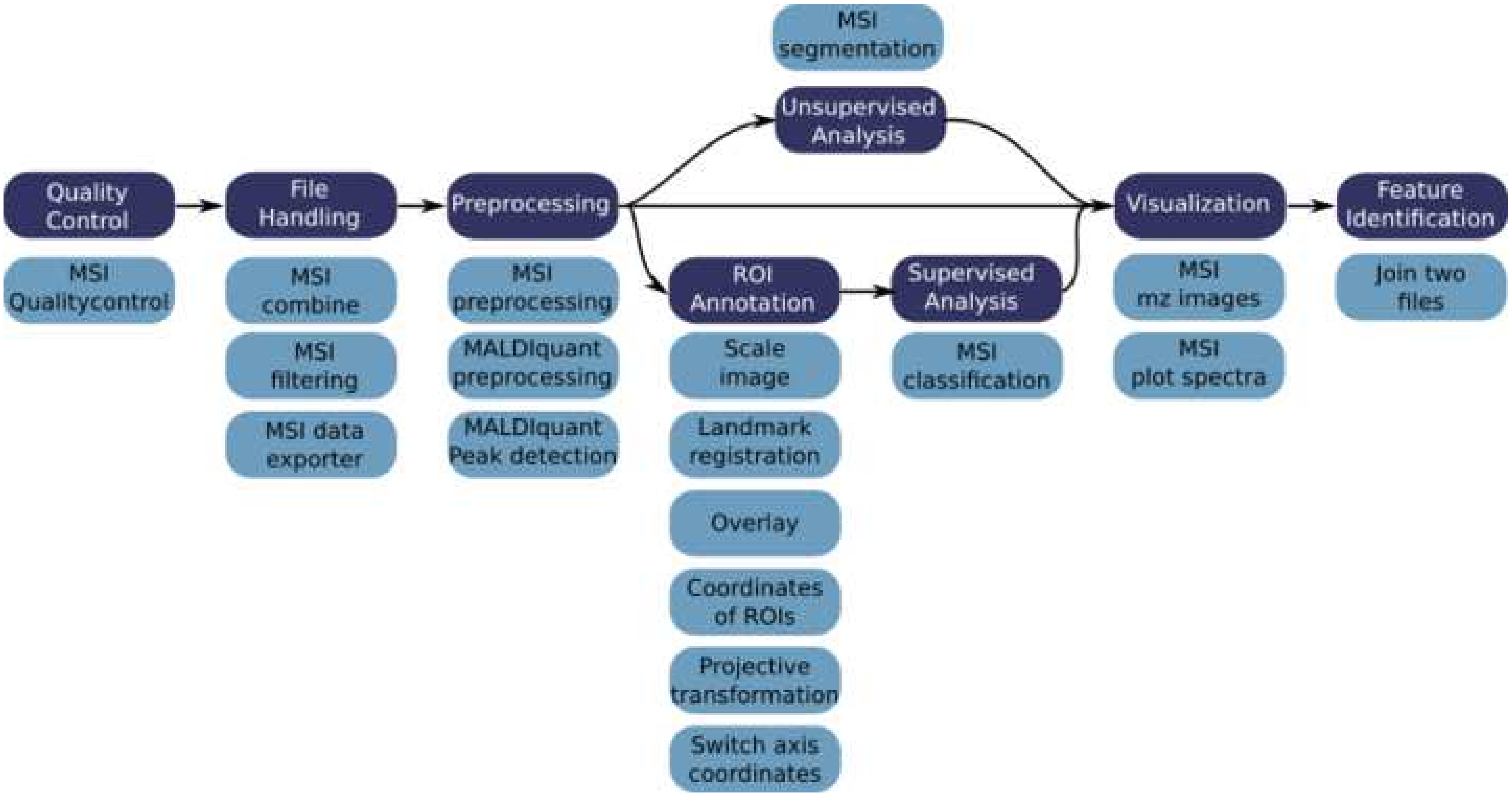

Figure

Click here to access/download;Figure;Figure2.png

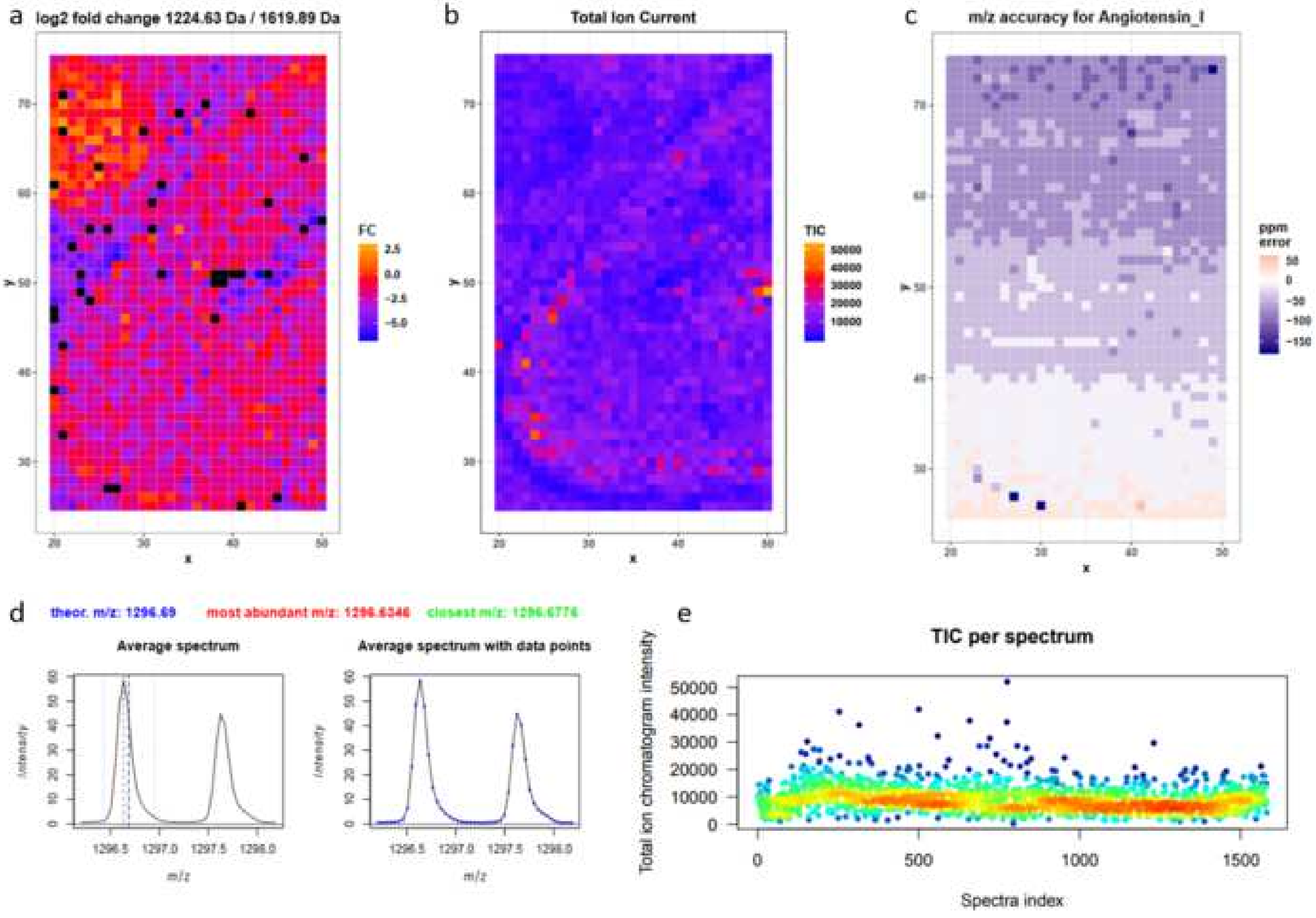

Figure
Click here to access/download;Figure;Figure3.png

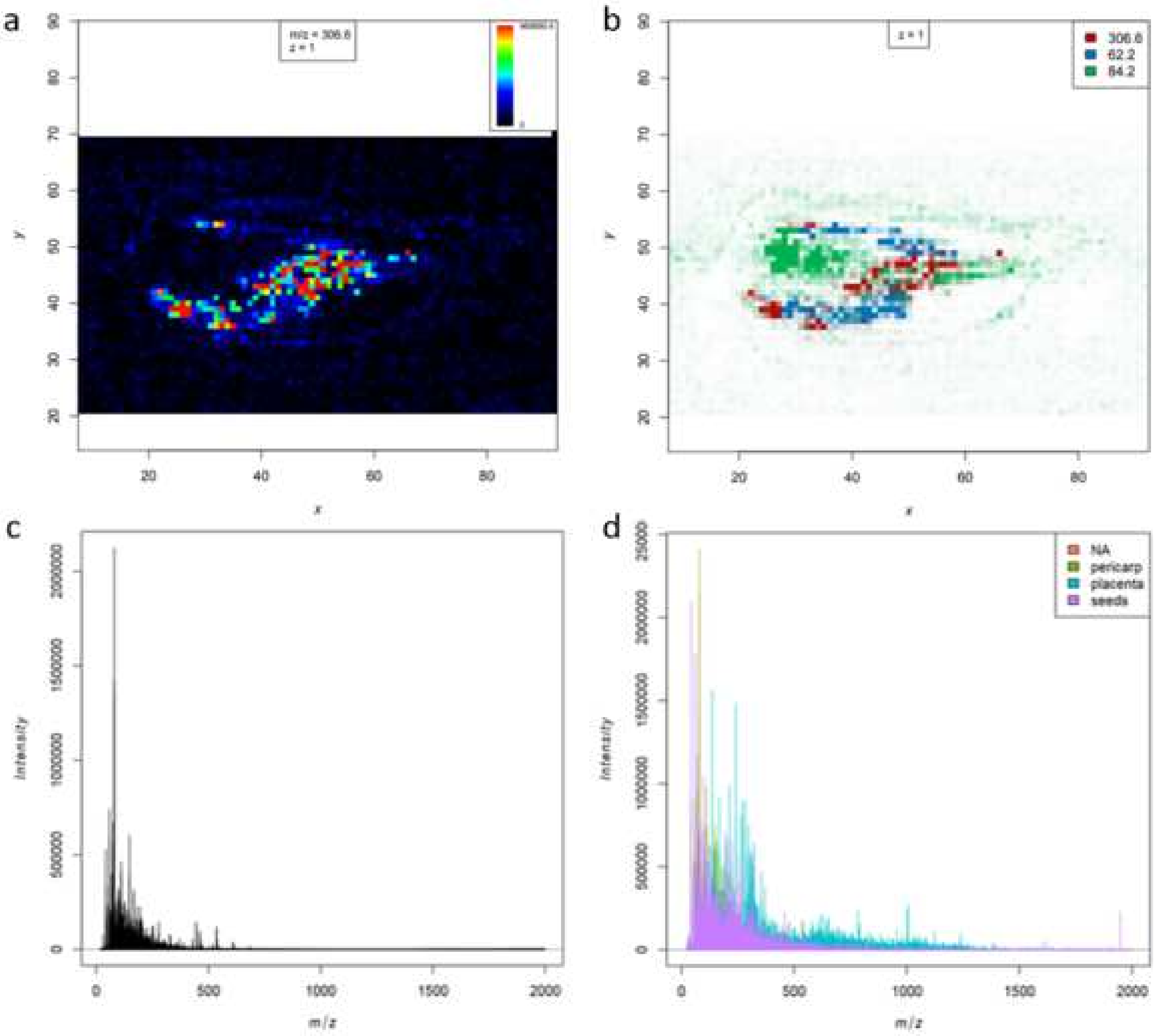

Figure

Click here to access/download;Figure;Figure4pca.png

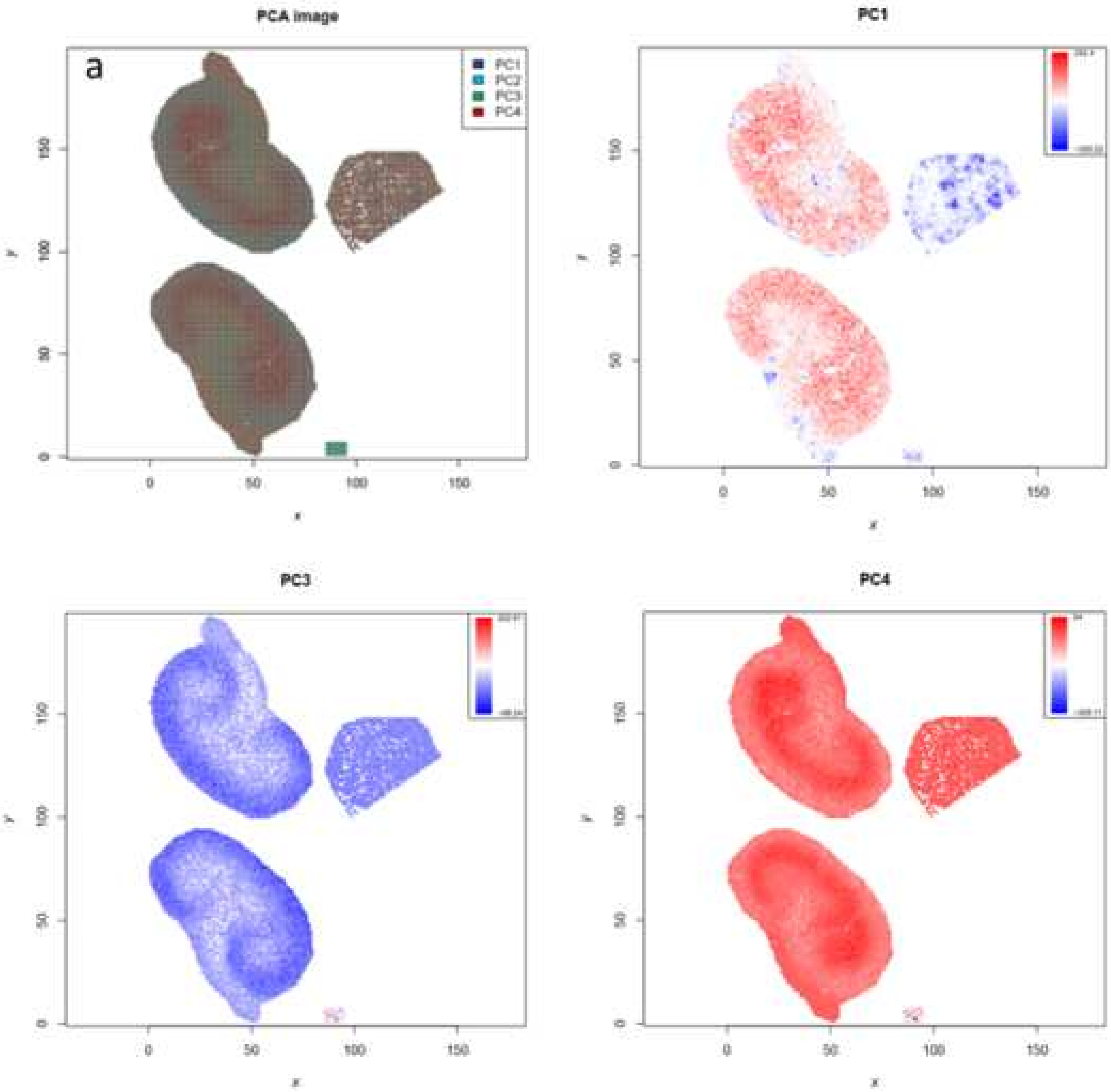

Click here to access/download;Figure;Figure5.png

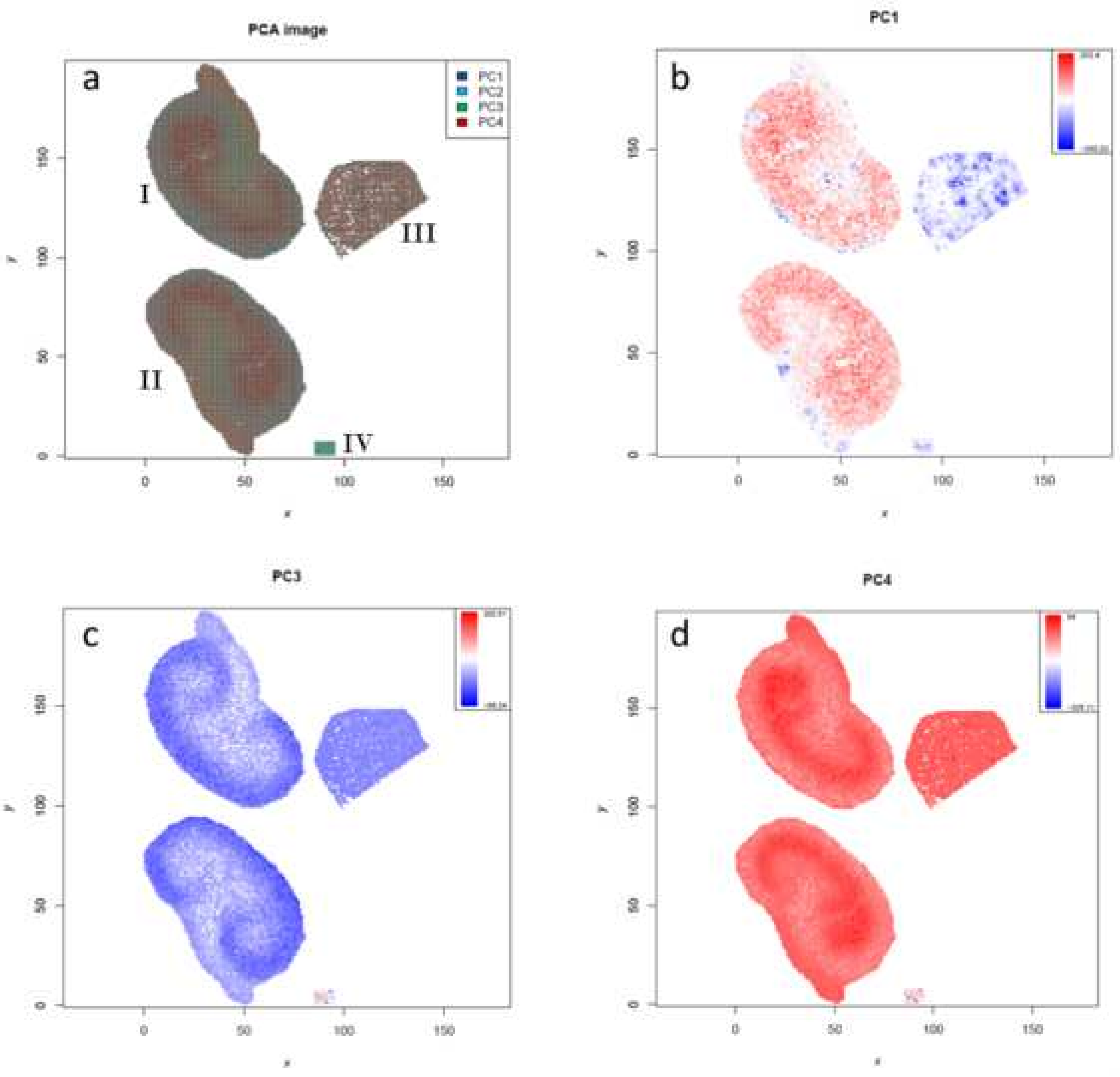

Figure

Click here to access/download;Figure;Figure6.png

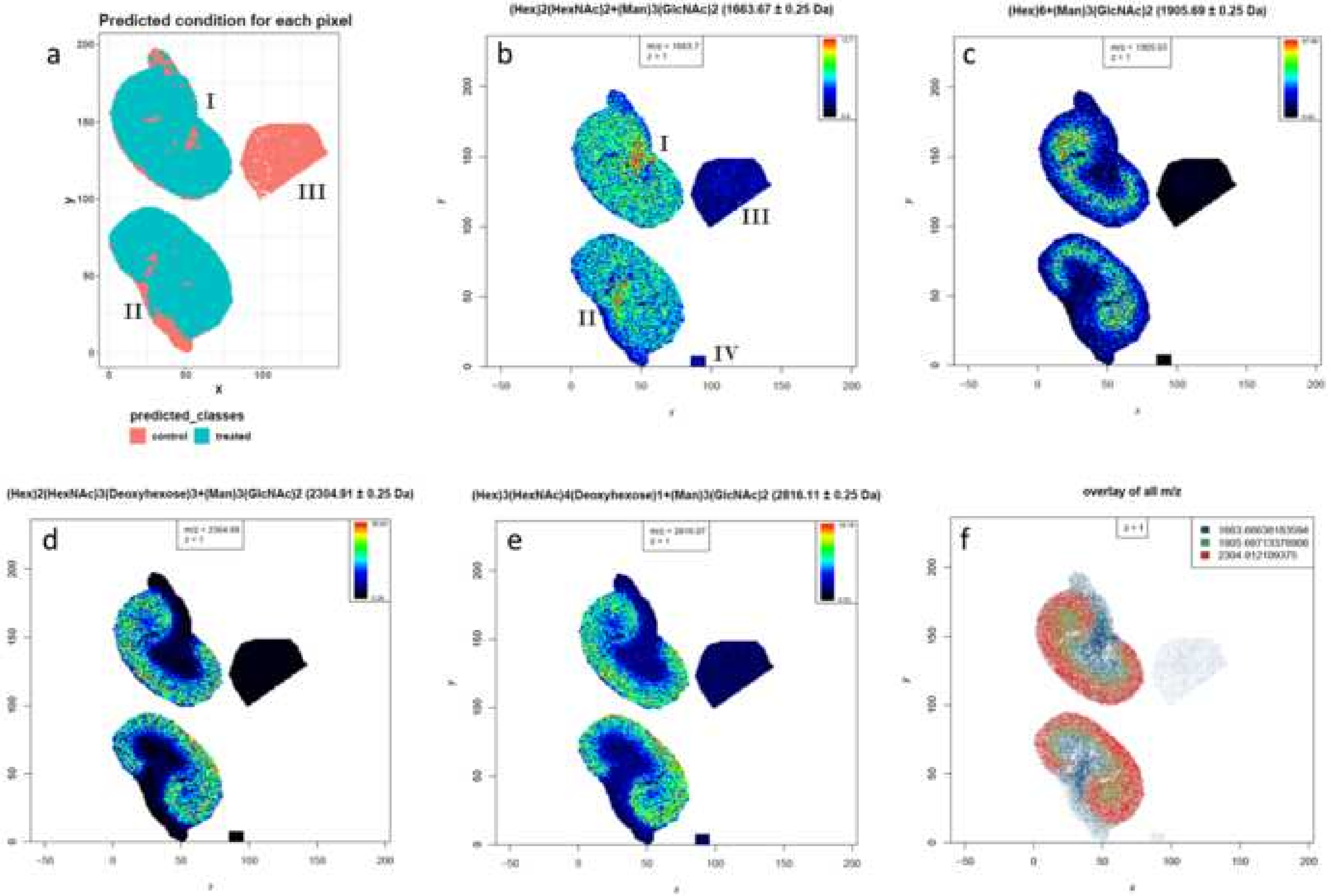

Click here to access/download
**Supplementary Material**
Additional file 1.xlsx

Click here to access/download
**Supplementary Material**
Additional file 2.xlsx

Click here to access/download
**Supplementary Material**
Additional file 3.xlsx

Click here to access/download
**Supplementary Material**
Additional file 4.pdf
