# Supplementary material for: Accessible and reproducible mass spectrometry imaging data analysis in Galaxy
Source: Gigascience. 2019 Dec 9;8(12):giz143. doi: 10.1093/gigascience/giz143 (PMC6901077; doi:10.1093/gigascience/giz143)
Supplement: giz143_Supplemental_Additional_Files [file giz143_supplemental_additional_files.zip › Additional file 4.pdf]

Additional File 4: Exemplary quality control plots for the combined N-glycan imaging file

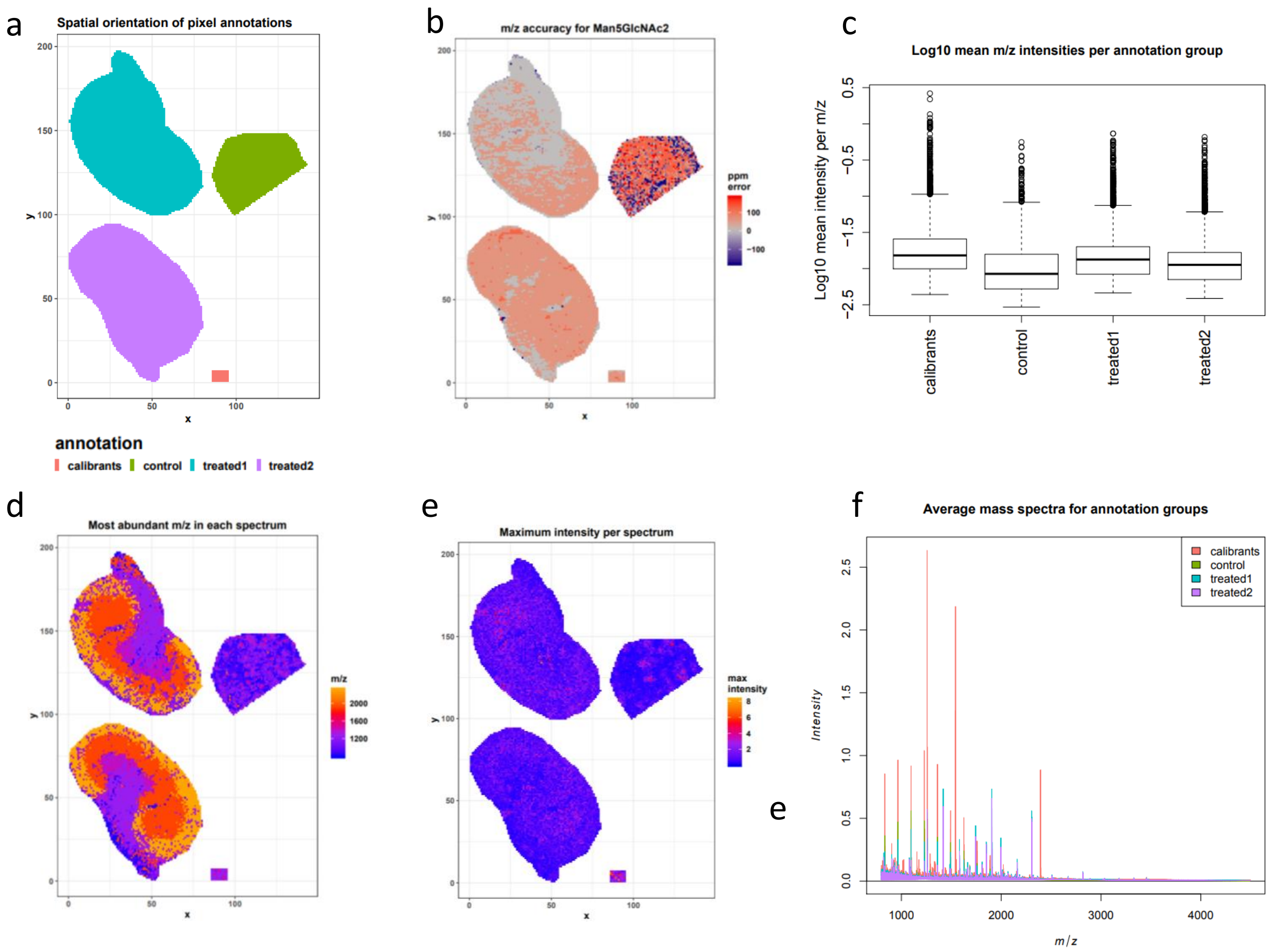

The four files were combined into one file and inspected with the MSI qualitycontrol tool. a) Pixel annotations b) m/z accuracy for the N-glycan that was used as calibrant in the calibrant file c) distribution of mean intensities for each m/z feature and annotation group d) Most abundant m/z feature in each spectrum e) Maximum intensity in each spectrum f) Average mass spectra for each annotation group.
