# Supplementary material for: Accessible and reproducible mass spectrometry imaging data analysis in Galaxy
Source: Gigascience. 2019 Dec 9;8(12):giz143. doi: 10.1093/gigascience/giz143 (PMC6901077; doi:10.1093/gigascience/giz143)
Supplement: giz143_Response_to_Reviewer_Comments_Original_Submission [file giz143_response_to_reviewer_comments_original_submission.pdf]

Author's Response To Reviewer Comments

Close

We sincerely thank the reviewers for their thoughtful comments and inspections of our manuscript. We have improved the manuscript by carefully addressing the raised concerns and provide a point-by-point response below. In the manuscript, we have highlighted paragraphs with major changes in blue to allow an easier tracking of the changes.

Reviewer #1 comment 1:
This Technical Note describes software tools for mass spectrometry image (MSI) analysis and integration of these tools into Galaxy. This enables reproducible MSI analysis to be accomplished in the context of a graphical user interface. The manuscript is well written and the authors should be commended for ensuring that the software tools are all open source.
In Figure 1 the authors highlight all 18 open source software tools in the MSI analysis toolkit. However, in the manuscript detailed descriptions are only provided for 12 of the software tools. I would like to see descriptions for the following 6 software tools: 1) Scale image; 2) Landmark registration; 3) Overlay; 4) Coordinates of ROIs; 5) Projective transformation; 6) Switch axis coordinates. This would be of great benefit to the end user who may wish to reuse these software tools. It would be additionally helpful if these 6 tools were listed in the supplementary file (supp-1.xlsx), which currently only lists 12 Galaxy tools.
>> Authors:
We thank the reviewer for pointing out this inconsistency, which has now been corrected by adding descriptions of the imaging tools in the main text and also added their specifications in supplementary file 1.

Reviewer #1 comment 2:
In the section entitled "Accessibility & training" the authors state that, "all tools are deposited in the Galaxy Toolshed from where they can be easily installed into any other Galaxy instance". Could the authors please specify where in the Galaxy Toolshed I would find all 18 software tools? The link that the authors provide (https://toolshed.g2.bx.psu.edu/) does not bring me to a directory that lists all 18 tools. Furthermore, I cannot see any mass spectrometry tools listed on this page. A direct link to the 18 software tools would be far more helpful and would encourage reuse of the toolkit.
>> Authors:
We agree with the reviewer that direct toolshed links for each tool are more helpful. Therefore we added a supplementary file with the direct links for all 18 tools.
Reviewer #1 comment 3:
As proof-of-principle, the authors used a publicly available N-linked glycan imaging dataset as a test dataset. The authors acknowledged that annotation was "infeasible on the MSI images" and they describe a method whereby the researcher annotates an adjacent histological section and then co-registers the two images using an affine transform. I was intrigued by this approach but I was unable to find the resolution of the MS images in the manuscript. Furthermore, the authors did not provide any detail of pixel size, or even scale bars, for the murine kidney test data set that is shown in Figures 2 and 3. However, I did find these details in the original publications that describe these data where it is stated that MSI profiling utilised a "centre to centre spacing of 250 µm". (Gustafsson et al., 2018, Data Brief. 21: 185-188; Gustafsson et al., 2015, Anal Bioanal Chem. 407(8): 2127-2139). Should we infer from this that the pixel dimensions are approximately 250µm x 250µm? I would like some clarity from the authors on the resolution of the MS images.
>> Authors:

We sincerely apologize for not being clear in the spatial resolution of the N-glycan dataset. We have extended the text to address the issue. The PNGase F was spotted with 250µm centre to centre spacing but the data was acquired by oversampling with a spatial resolution of 100µm leading to a pixel size of 100µm (Reference Gustafsson et al. 2015, page 2130).

Reviewer #1 comment 4:
Whereas I can see great value in the software tools, I would question the utility of this low-resolution

MSI dataset. A pixel resolution of 250µm x 250µm is fairly coarse. Consequently, it would be impossible to determine from these data whether the MS profiles reflect N-linked glycan distribution in key microanatomical compartments of the kidney such as the proximal tubule, juxtaglomerular apparatus, or podocyte. I was wondering whether the authors have used their software tools on cellular-resolution MSI data? From a biological perspective, this would be far more interesting. In this respect, the NIH/NIDDK-funded GUDMAP Consortium have delivered a whole series of publications that detail gene expression in the kidney at the microanatomical level (Brunskill et al., 2008, Dev Cell. 15(5):781-91; Lindström et al., 2018, J Am Soc Nephrol. 29(3):785-805; Adam et al., 2017, Development. 144(19):3625-3632). I was wondering whether the authors would consider using their toolkit to analyse more biologically relevant, single-cell resolution MSI datasets? This would be of greater interest to the research community and would be an improved means of highlighting the reuse potential of this toolkit.
>> Authors:
We fully understand the concerns of the reviewer but have to apologize that commercial mass spectrometers and available protocols do not yet allow for single cell analysis. The reviewer refers to gene expression dataset with single-cell resolution. However, such datasets do not exists in the field of MSI; a limitation that is due to technical shortcomings of the method. Technological progress may remove this limitation in the future, but as of today, most MSI experiments are performed with a spatial resolution between 30 and 250 µm. The actual resolution is dependent on the purpose of the experiment and limited by the technical capabilities of the mass spectrometer and the limited amount of analytes that can be extracted from a single cell. While the technical improvements aim towards enabling cellular resolution, data analysis steps are mainly working on single pixel (mass spectra) and therefore only affected by the increasing file size as more pixels will be measured to cover the same sample area. Therefore, the limitation for single cell analysis lies not in the analysis tools but in the method itself. To show the usefulness of our tools, we also re-analyze volatile metabolites in a chilli section and the peptide distribution in a mouse kidney; both are part of the accompanying training material. Hence, we respectfully decline further case study analysis.

Reviewer #2 comment 1:
The authors have integrated three different mass spectrometry imaging (MSI) software packages (Cardinal, MALDIquant, and scikit-image) into the Galaxy framework. The Galaxy tools and workflows presented cover a wide range of common MSI data processing and analysis steps, and allow Proteomics and Metabolomics researchers to build useful and flexible workflows. It is evident that a substantial amount of work has gone not just into wrapping the different MSI packages into the Galaxy Framework, but also in developing and writing the online training material and use cases. The manuscript is well written and is relevant to the scope of the journal. The manuscript could benefit from revision as indicated in the detailed comments listed below.
Page 4 - "Galaxy is used by hundred thousands of researchers and provides thousands of different tools for many different scientific fields." - Include more realistic and accurate statistics on users and tools. The reference cited [25, Afgan et al.] does provide some specific numbers / statistics on tools and users.
>> Authors:

We did not provide specific numbers for two reasons: 1) The specific numbers do not reflect the number of researchers using Galaxy, as they only refer to the main Galaxy server, neglecting the european, australian and the many smaller servers. 2) The specific numbers were collected about one year ago and are already outdated and will be even more outdated when the manuscript will be read in the future.
Reviewer #2 comment 2:
Page 6 - Change "All tools are deliberately build" to "all tools are deliberately built"
>> Authors:
Done as suggested.

Reviewer #2 comment 3:
Page 6 – Change "cleverly combining" to "combining"
>> Authors:
Done as suggested.

Reviewer #2 comment 4:
Page 6 - Please, add a short paragraph to section "The newly available MSI toolset in the Galaxy Framework" that described the status of the different packages (i.e. Cardinal, MALDIquant, etc) - for

example, are they all commonly used tools? Well established? Cutting edge?
>> Authors:
Done as suggested.

Reviewer #2 comment 5:
Page 7 - "Nowadays" too informal and not specific - past n years? Add a reference to back up statement.
>> Authors:

We changed the sentence accordingly and reference 43 lists current vendor imzML compatible software and imzML converting software.

Reviewer #2 comment 6:
Page 7 - "file collections" - Should this be "dataset collections" (i.e. "official" galaxy terminology)? If so, change throughout the manuscript.
>> Authors:
Done as suggested.

Reviewer #2 comment 7:
Page 7 - "Numerous files can be represented in a file collection allowing simultaneous analysis of all files while the effort for the user is the same as for a single file". This is somewhat misleading. Whilst dataset collections can be very useful in Galaxy and in some cases allow parallel processing to be performed, in an optimised or user friendly manner, they often require an additional step to prepare or merge the data.
>> Authors:

That's true, our sentence was misleading. We changed it into "while the effort for the user is similar as for single files".
Reviewer #2 comment 8:
Page 7 - Add more information on imzML format for the reader
>> Authors:

As the imzML format is the community standard, we thought this information is not useful in the manuscript. The file information that is relevant for successfully handling this file type are described in more detail in the training material.

Reviewer #2 comment 9:
Page 8: Change "Spectra with bad quality" to "poor quality spectra"
>> Authors:
Done as suggested.

Reviewer #2 comment 10:
Page 10 - "However, annotation of these ROIs is infeasible on the MSI images" explain why
>> Authors:
Done as suggested.

Reviewer #2 comment 11:
Page 10 - Add a reference for "Random sample consensus (RANSAC)"
>> Authors:
Done as suggested.

Reviewer #2 comment 12:
Page 11 - "Join two files on a column allowing a small difference:" - Is this the real name of the tools. Convoluted name for a tool.
>> Authors:

Yes, it's the full name, we thought that it's better to be descriptive than short for our users.

Reviewer #2 comment 13:
Page 14 - Change "Matching those features that potentially are N-glycans to the m/z list of the original publication" to "mapping features to N-glycans reported in the original publication"
>> Authors:
Done as suggested.

Reviewer #2 comment 14:
Page 14 - Change "While we missed the 1647.635 m/z N-glycan, we found another N-glycan with 1542.62 m/z." to "whilst our workflow did not identify the reported N-glycan at 1647.635 m/z, an additional N-glycan at 1542.62 m/z was found."
>> Authors:
Done as suggested.

Reviewer #2 comment 15:
Page 16 - "In Gustafsson's own terms from a recent publication, our results show that their results are reproducible, because we, as another group, have followed as closely as possible their data analysis procedure and arrived at similar results [16].The reproducibility of the results shows the capacity of our pipeline. To enable what Gustafsson has described as "methods reproducibility" we provide the complete analysis history and the corresponding workflow. With this in hand, any other researcher can use the same tools and parameters in Galaxy to obtain the same result as we did." Overly wordy, therefore rephrase.
>> Authors:
We agree that this paragraph was confusing, therefore we rewrote it to make it easier to read and understand.

Reviewer #2 comment 16:
Page 17 - "Publishing histories and workflows from Galaxy requires only a few clicks" - too informal. Rephrase, in particular the words "a few clicks".
>> Authors:
We changed the sentence accordingly.

Reviewer #2 comment 17:
Preprocessing (Page 10-14)
Provide a short description for TIC normalisation, baseline removal and smoothing, etc, including details for the different parameters used (Supplementary information),? I.e. which algorithms and parameters for peak picking, detection of monoisotopic peaks and binning were applied? And why?
>> Authors:

The N-glycan dataset that was used to demonstrate a use-case for the workbench is also part of the Galaxy training network (https://training.galaxyproject.org/training-material/topics/metabolomics/tutorials/msi-finding-nglycans/tutorial.html).
In this tutorial we describe in detail the different analysis steps and the choice of algorithms and parameters. The overall goal of the case study was to reproduce the original study, therefore we have chosen algorithms and parameters that most closely resembled the data analysis from the original study. A link to the training material repository is included in the manuscript, which we think is more sustainable, as we plan to extend and update the training material in the future.
For users that are interested in more details of the case study, we have published a real-world Galaxy history which provides all details about used algorithms, parameters, tool version and references.
The authors would like to stress the fact, that we think that sharing a history is more reproducible and transparent than trying to describe parameters in text.

Reviewer #2 comment 18:
Add a table that summarises the different options for each pre-processing step. What normalization and peak picking options are available? I.e. "multitude of algorithms". Provide a more detailed summary. This could be a further advantage over proprietary software having more flexibility on pre-processing steps.
>> Authors:

We added this information as additional file.

Reviewer #2 comment 19:
Is median fold change used (or available as an option) as described in the following paper? Abbassi-Ghadi, Nima, Emrys A Jones, Kirill A Veselkov, Juzheng Huang, Sacheen Kumar, Nicole Strittmatter, Ottmar Golf, et al. 2015. "Repeatability and Reproducibility of Desorption Electrospray Ionization-Mass Spectrometry (DESI-MS) for the Imaging Analysis of Human Cancer Tissue: A Gateway for Clinical

Applications." Analytical Methods 7 (1): 71-80. https://doi.org/10.1039/C4AY01770F.
>> Authors:

Unfortunately, no. Not yet. For now we have focused on the implementation of Cardinal and MALDIquant functionalities that contain the most common normalization methods TIC and median. We plan to extend and update our tools if requested by users and are happy to add additional normalization methods. We encourage everyone to use the normal Galaxy communication channels like gitter and help.galaxyproject.org to get in touch with us and provide feedback.

Reviewer #2 comment 20:
Include references for the methods/approached that have been applied. For example - A detailed description for the underlying principles applied as part of MALDIquant pre-processing and MALFIquant peak detection are missing. Add the relevant references and details (add to Supplementary Information if needed). Same applies to other methods / approaches.
>> Authors:
Thanks for this recommendation. We added further references to the manuscript whenever possible. Unfortunately most of the Cardinal functions are specific methods for the Cardinal package and no further descriptions does exist except for the code in the github repository which is referenced already in our manuscript.

Reviewer #2 comment 21:
Add figures to the main text or Supplementary Information for the following:
Page 8 - Figures in the data visualization section would make for a more intuitive read - For instance - include figures for a number of the "30 different plots"
>> Authors:
Done as suggested.

Reviewer #2 comment 22:
Page 8 - Include figures for both the MSI image and MSI plot spectra (with overlay)
>> Authors:
Done as suggested.

Reviewer #2 comment 23:
Page 9 - ROI annotation tool, particularly use of "affine transformation", could be aided by a figure
>> Authors:
Done as suggested.

Reviewer #2 comment 24:
Page 14 - Outputs / Results "Quality Control" tool
>> Authors:
Done as suggested.

Reviewer #3 comment 1:
This is an interesting paper that provides new tools for the Galaxy platform and that will be of use to biomedical researchers generating and analysing MSI data. The authors demonstrate the value of the toolset that they have developed by reanalysing a published dataset and they provide open access to source code and additional training documentation. Developing open source tools is commendable and I therefore support publication.
Minor Revisions:
Section describing the new MSI toolset: Making more of a distinction in the text between the newly developed tools and existing Galaxy tools would be helpful to the reader.
>> Authors:

We agree with the reviewer and have made it more clear in the manuscript to distinguish between new tools and already existing tools. Moreover, the newly developed tools are depicted in Figure 1.
Reviewer #3 comment 2:
I also suggest adding 'tool(s)' to each subheading within this section i.e. 'Quality control and visualization tools:'
>> Authors:
Done as suggested.

Reviewer #3 comment 3:
Figure 1: The figure title should make it clear that these are the newly developed Galaxy tools: the use of 'associated' is ambiguous.
>> Authors:
Done as suggested.

Reviewer #3 comment 4:
The figure legend could be improved by clarifying that the MSI data analysis steps are in dark blue and the new tools are shown in pale blue.
>> Authors:
Done as suggested.

Reviewer #3 comment 5:
Sectioning describing Accessibility and training: I found navigation to the training sets difficult. The link to the Galaxy Training Network/repository is to the home page and the reader then has to click through several pages to find each of the 3 examples. The names are not obvious from the manuscript and I had to click through a number of other examples to find them. The training material is excellent but just difficult to find.
>> Authors:
We added the information next to the link to the training repository: mass spectrometry imaging tutorials can be found in the metabolomics and proteomics categories of the training material.

Reviewer #3 comment 6:
Availability of supporting data: I did not seem to have the necessary permission to access the datasets provided in the last link.
>> Authors:
We are sorry for the inconvenience. Galaxy values data privacy a lot and we made a mistake in granting everyone permissions. We updated the dataset permissions to make sure that the link works now.

Reviewer #3 comment 7:
Add FAIR to the list of abbreviations.
>> Authors:
Done as suggested.

Close
