# Supplementary material for: Accessible and reproducible mass spectrometry imaging data analysis in Galaxy
Source: Gigascience. 2019 Dec 9;8(12):giz143. doi: 10.1093/gigascience/giz143 (PMC6901077; doi:10.1093/gigascience/giz143)
Supplement: giz143_Reviewer_1_Report_Original_Submission — Chris Armit -- 5/27/2019 Reviewed [file giz143_reviewer_1_report_original_submission.pdf]

Reviewer Report

Title: Accessible and reproducible mass spectrometry imaging data analysis in Galaxy

Version: Original Submission **Date:** 5/27/2019

Reviewer name: Chris Armit

Reviewer Comments to Author:

This Technical Note describes software tools for mass spectrometry image (MSI) analysis and integration of these tools into Galaxy. This enables reproducible MSI analysis to be accomplished in the context of a graphical user interface. The manuscript is well written and the authors should be commended for ensuring that the software tools are all open source.

Major comments

In Figure 1 the authors highlight all 18 open source software tools in the MSI analysis toolkit. However, in the manuscript detailed descriptions are only provided for 12 of the software tools. I would like to see descriptions for the following 6 software tools: 1) Scale image; 2) Landmark registration; 3) Overlay; 4) Coordinates of ROIs; 5) Projective transformation; 6) Switch axis coordinates. This would be of great benefit to the end user who may wish to reuse these software tools. It would be additionally helpful if these 6 tools were listed in the supplementary file (supp-1.xlsx), which currently only lists 12 Galaxy tools.

In the section entitled "Accessibility & training" the authors state that, "all tools are deposited in the Galaxy Toolshed from where they can be easily installed into any other Galaxy instance". Could the authors please specify where in the Galaxy Toolshed I would find all 18 software tools? The link that the authors provide (https://toolshed.g2.bx.psu.edu/) does not bring me to a directory that lists all 18 tools. Furthermore, I cannot see any mass spectrometry tools listed on this page. A direct link to the 18 software tools would be far more helpful and would encourage reuse of the toolkit.

As proof-of-principle, the authors used a publicly available N-linked glycan imaging dataset as a test dataset. The authors acknowledged that annotation was "infeasible on the MSI images" and they describe a method whereby the researcher annotates an adjacent histological section and then co-registers the two images using an affine transform. I was intrigued by this approach but I was unable to find the resolution of the MS images in the manuscript. Furthermore, the authors did not provide any detail of pixel size, or even scale bars, for the murine kidney test data set that is shown in Figures 2 and 3. However, I did find these details in the original publications that describe these data where it is stated that MSI profiling utilised a "centre to centre spacing of 250â€¯Âµm". (Gustafsson et al., 2018, Data Brief. 21: 185-188; Gustafsson et al., 2015, Anal Bioanal Chem. 407(8): 2127-2139). Should we infer from this that the pixel dimensions are approximately 250Âµm x 250Âµm? I would like some clarity from the authors on the resolution of the MS images.

Whereas I can see great value in the software tools, I would question the utility of this low-resolution MSI dataset. A pixel resolution of 250Âµm x 250Âµm is fairly coarse. Consequently, it would be impossible to determine from these data whether the MS profiles reflect N-linked glycan distribution in key microanatomical compartments of the kidney such as the proximal tubule, juxtaglomerular

apparatus, or podocyte. I was wondering whether the authors have used their software tools on cellular-resolution MSI data? From a biological perspective, this would be far more interesting. In this respect, the NIH/NIDDK-funded GUDMAP Consortium have delivered a whole series of publications that detail gene expression in the kidney at the microanatomical level (Brunskill et al., 2008, Dev Cell. 15(5):781-91; LindstrÃ¶m et al., 2018, J Am Soc Nephrol. 29(3):785-805; Adam et al., 2017, Development. 144(19):3625-3632). I was wondering whether the authors would consider using their toolkit to analyse more biologically relevant, single-cell resolution MSI datasets? This would be of greater interest to the research community and would be an improved means of highlighting the reuse potential of this toolkit.

Level of Interest

Please indicate how interesting you found the manuscript: Choose an item.

Quality of Written English

Please indicate the quality of language in the manuscript: Choose an item.

Declaration of Competing Interests

Please complete a declaration of competing interests, considering the following questions:

- Have you in the past five years received reimbursements, fees, funding, or salary from an organisation that may in any way gain or lose financially from the publication of this manuscript, either now or in the future?
- Do you hold any stocks or shares in an organisation that may in any way gain or lose financially from the publication of this manuscript, either now or in the future?
- Do you hold or are you currently applying for any patents relating to the content of the manuscript?
- Have you received reimbursements, fees, funding, or salary from an organization that holds or has applied for patents relating to the content of the manuscript?
- Do you have any other financial competing interests?
- Do you have any non-financial competing interests in relation to this paper?

If you can answer no to all of the above, write 'I declare that I have no competing interests' below. If your reply is yes to any, please give details below.

I declare that I have no competing interests.

I agree to the open peer review policy of the journal. I understand that my name will be included on my report to the authors and, if the manuscript is accepted for publication, my named report including any

attachments I upload will be posted on the website along with the authors' responses. I agree for my report to be made available under an Open Access Creative Commons CC-BY license (http://creativecommons.org/licenses/by/4.0/). I understand that any comments which I do not wish to be included in my named report can be included as confidential comments to the editors, which will not be published.

Choose an item.

To further support our reviewers, we have joined with Publons, where you can gain additional credit to further highlight your hard work (see: https://publons.com/journal/530/gigascience). On publication of this paper, your review will be automatically added to Publons, you can then choose whether or not to claim your Publons credit. I understand this statement.

Yes Choose an item.
