# Supplementary material for: Accessible and reproducible mass spectrometry imaging data analysis in Galaxy
Source: Gigascience. 2019 Dec 9;8(12):giz143. doi: 10.1093/gigascience/giz143 (PMC6901077; doi:10.1093/gigascience/giz143)
Supplement: giz143_Reviewer_2_Report_Original_Submission — Ralph Weber -- 6/4/2019 Reviewed [file giz143_reviewer_2_report_original_submission.pdf]

Reviewer Report

Title: Accessible and reproducible mass spectrometry imaging data analysis in Galaxy

Version: Original Submission **Date:** 6/4/2019

Reviewer name: Ralph Weber

Reviewer Comments to Author:

The authors have integrated three different mass spectrometry imaging (MSI) software packages (Cardinal, MALDIquant, and scikit-image) into the Galaxy framework. The Galaxy tools and workflows presented cover a wide range of common MSI data processing and analysis steps, and allow Proteomics and Metabolomics researchers to build useful and flexible workflows. It is evident that a substantial amount of work has gone not just into wrapping the different MSI packages into the Galaxy Framework, but also in developing and writing the online training material and use cases. The manuscript is well written and is relevant to the scope of the journal. The manuscript could benefit from revision as indicated in the detailed comments listed below.
Revisions:
Page 4 - "Galaxy is used by hundred thousands of researchers and provides thousands of different tools for many different scientific fields." - Include more realistic and accurate statistics on users and tools. The reference cited [25, Afgan et al.] does provide some specific numbers / statistics on tools and users.
Page 6 - Change "All tools are deliberately build" to "all tools are deliberately built"
Page 6 - Change"cleverly combining" to "combining"
Page 6 - Please, add a short paragraph to section "The newly available MSI toolset in the Galaxy Framework" that described the status of the different packages (i.e. Cardinal, MALDIquant, etc) - for example, are they all commonly used tools? Well established? Cutting edge?
Page 7 - "Nowadays" too informal and not specific - past n years? Add a reference to back up statement.
Page 7 - "file collections" - Should this be "dataset collections" (i.e. "official" galaxy terminology)? If so, change throughout the manuscript.
Page 7 - "Numerous files can be represented in a file collection allowing simultaneous analysis of all files while the effort for the user is the same as for a single file"
This is somewhat misleading. Whilst dataset collections can be very useful in Galaxy and in some cases allow parallel processing to be performed, in an optimised or user friendly manner, they often require an additional step to prepare or merge the data.
Page 7 - Add more information on imzML format for the reader
Page 8: Change "Spectra with bad quality" to "poor quality spectra"
Page 10 - "However, annotation of these ROIs is infeasible on the MSI images" explain why
Page 10 - Add a reference for "Random sample consensus (RANSAC)"
Page 11 - "Join two files on a column allowing a small difference:" - Is this the real name of the tools. Convoluted name for a tool.
Page 14 - Change "Matching those features that potentially are N-glycans to the m/z list of the original publication" to "mapping features to N-glycans reported in the original publication"

Page 14 - Change "While we missed the 1647.635 m/z N-glycan, we found another N-glycan with 1542.62 m/z." to "whilst our workflow did not identify the reported N-glycan at 1647.635 m/z, an additional N-glycan at 1542.62 m/z was found."

Page 16 - "In Gustafsson's own terms from a recent publication, our results show that their results are reproducible, because we, as another group, have followed as closely as possible their data analysis procedure and arrived at similar results [16].The reproducibility of the results shows the capacity of our pipeline. To enable what Gustafsson has described as "methods reproducibility" we provide the complete analysis history and the corresponding workflow. With this in hand, any other researcher can use the same tools and parameters in Galaxy to obtain the same result as we did." Overly wordy, therefore rephrase.

Page 17 - "Publishing histories and workflows from Galaxy requires only a few clicks" - too informal. Rephrase, in particular the words "a few clicks".

Preprocessing (Page 10-14)

- Provide a short description for TIC normalisation, baseline removal and smoothing, etc, including details for the different parameters used (Supplementary information),? I.e. which algorithms and parameters for peak picking, detection of monoisotopic peaks and binning were applied? And why?
- Add a table that summarises the different options for each pre-processing step.
- What normalization and peak picking options are available? I.e. "multitude of algorithms". Provide a more detailed summary.
    - This could be a further advantage over proprietary software having more flexibility on pre-processing steps
- Is median fold change used (or available as an option) as described in the following paper?
    - Abbassi-Ghadi, Nima, Emrys A Jones, Kirill A Veselkov, Juzheng Huang, Sacheen Kumar, Nicole Strittmatter, Ottmar Golf, et al. 2015. "Repeatability and Reproducibility of Desorption Electrospray Ionization-Mass Spectrometry (DESI-MS) for the Imaging Analysis of Human Cancer Tissue: A Gateway for Clinical Applications." Analytical Methods 7 (1): 71-80. https://doi.org/10.1039/C4AY01770F. o
- Include references for the methods/approached that have been applied. For example - A detailed description for the underlying principles applied as part of MALDIquant pre-processing and MALFIquant peak detection are missing. Add the relevant references and details (add to Supplementary Information if needed). Same applies to other methods / approaches.

Add figures to the main text or Supplementary Information for the following:

Page 8 - Figures in the data visualization section would make for a more intuitive read

- For instance - include figures for a number of the "30 different plots"

Page 8 - Include figures for both the MSI image and MSI plot spectra (with overlay)

Page 9 - ROI annotation tool, particularly use of "affine transformation", could be aided by a figure

Page 14 - Outputs / Results "Quality Control" tool

Level of Interest

Please indicate how interesting you found the manuscript: Choose an item.

Quality of Written English

Please indicate the quality of language in the manuscript: Choose an item.

Declaration of Competing Interests

Please complete a declaration of competing interests, considering the following questions:

- Have you in the past five years received reimbursements, fees, funding, or salary from an organisation that may in any way gain or lose financially from the publication of this manuscript, either now or in the future?
- Do you hold any stocks or shares in an organisation that may in any way gain or lose financially from the publication of this manuscript, either now or in the future?
- Do you hold or are you currently applying for any patents relating to the content of the manuscript?
- Have you received reimbursements, fees, funding, or salary from an organization that holds or has applied for patents relating to the content of the manuscript?
- Do you have any other financial competing interests?
- Do you have any non-financial competing interests in relation to this paper?

If you can answer no to all of the above, write 'I declare that I have no competing interests' below. If your reply is yes to any, please give details below.

I declare that I have no competing interests

I agree to the open peer review policy of the journal. I understand that my name will be included on my report to the authors and, if the manuscript is accepted for publication, my named report including any attachments I upload will be posted on the website along with the authors' responses. I agree for my report to be made available under an Open Access Creative Commons CC-BY license (http://creativecommons.org/licenses/by/4.0/). I understand that any comments which I do not wish to be included in my named report can be included as confidential comments to the editors, which will not be published.

Choose an item.

To further support our reviewers, we have joined with Publons, where you can gain additional credit to further highlight your hard work (see: https://publons.com/journal/530/gigascience). On publication of this paper, your review will be automatically added to Publons, you can then choose whether or not to claim your Publons credit. I understand this statement.

Yes Choose an item.
