# Supplementary material for: Accessible and reproducible mass spectrometry imaging data analysis in Galaxy
Source: Gigascience. 2019 Dec 9;8(12):giz143. doi: 10.1093/gigascience/giz143 (PMC6901077; doi:10.1093/gigascience/giz143)
Supplement: giz143_Reviewer_3_Report_Original_Submission — Jane Armstrong -- 6/13/2019 Reviewed [file giz143_reviewer_3_report_original_submission.pdf]

Reviewer Report

Title: Accessible and reproducible mass spectrometry imaging data analysis in Galaxy

Version: Original Submission **Date:** 6/13/2019

Reviewer name: Jane Armstrong

Reviewer Comments to Author:

This is an interesting paper that provides new tools for the Galaxy platform and that will be of use to biomedical researchers generating and analysing MSI data. The authors demonstrate the value of the toolset that they have developed by reanalysing a published dataset and they provide open access to source code and additional training documentation. Developing open source tools is commendable and I therefore support publication.

Minor Revisions:

Section describing the new MSI toolset: Making more of a distinction in the text between the newly developed tools and existing Galaxy tools would be helpful to the reader. I also suggest adding 'tool(s)' to each subheading within this section i.e. 'Quality control and visualization tools:'

Figure 1: The figure title should make it clear that these are the newly developed Galaxy tools: the use of 'associated' is ambiguous. The figure legend could be improved by clarifying that the MSI data analysis steps are in dark blue and the new tools are shown in pale blue.

Sectioning describing Accessibility and training: I found navigation to the training sets difficult. The link to the Galaxy Training Network/repository is to the home page and the reader then has to click through several pages to find each of the 3 examples. The names are not obvious from the manuscript and I had to click through a number of other examples to find them. The training material is excellent but just difficult to find.

Availability of supporting data: I did not seem to have the necessary permission to access the datasets provided in the last link.

Add FAIR to the list of abbreviations.

Level of Interest

Please indicate how interesting you found the manuscript: Choose an item.

Quality of Written English

Please indicate the quality of language in the manuscript: Choose an item.

Declaration of Competing Interests

Please complete a declaration of competing interests, considering the following questions:

- Have you in the past five years received reimbursements, fees, funding, or salary from an organisation that may in any way gain or lose financially from the publication of this manuscript, either now or in the future?
- Do you hold any stocks or shares in an organisation that may in any way gain or lose financially from the publication of this manuscript, either now or in the future?
- Do you hold or are you currently applying for any patents relating to the content of the manuscript?
- Have you received reimbursements, fees, funding, or salary from an organization that holds or has applied for patents relating to the content of the manuscript?
- Do you have any other financial competing interests?
- Do you have any non-financial competing interests in relation to this paper?

If you can answer no to all of the above, write 'I declare that I have no competing interests' below. If your reply is yes to any, please give details below.

I declare that I have no competing interests

I agree to the open peer review policy of the journal. I understand that my name will be included on my report to the authors and, if the manuscript is accepted for publication, my named report including any attachments I upload will be posted on the website along with the authors' responses. I agree for my report to be made available under an Open Access Creative Commons CC-BY license (http://creativecommons.org/licenses/by/4.0/). I understand that any comments which I do not wish to be included in my named report can be included as confidential comments to the editors, which will not be published.

Choose an item.

To further support our reviewers, we have joined with Publons, where you can gain additional credit to further highlight your hard work (see: https://publons.com/journal/530/gigascience). On publication of this paper, your review will be automatically added to Publons, you can then choose whether or not to claim your Publons credit. I understand this statement.

Yes Choose an item.
